# INA complex liaises the F_1_F_o_-ATP synthase membrane motor modules

**DOI:** 10.1038/s41467-017-01437-z

**Published:** 2017-11-01

**Authors:** Nataliia Naumenko, Marcel Morgenstern, Robert Rucktäschel, Bettina Warscheid, Peter Rehling

**Affiliations:** 1 Department of Cellular Biochemistry, University Medical Center Göttingen, GZMB, D-37073 Göttingen, Germany; 2 Department of Biochemistry and Functional Proteomics, Faculty of Biology, University Freiburg, D-79104 Freiburg, Germany; 3 BIOSS Centre for Biological Signalling Studies, University of Freiburg, D-79104 Freiburg, Germany; 4 Max Planck Institute for Biophysical Chemistry, D-37077 Göttingen, Germany

## Abstract

The F_1_F_0_-ATP synthase translates a proton flux across the inner mitochondrial membrane into a mechanical rotation, driving anhydride bond formation in the catalytic portion. The complex’s membrane-embedded motor forms a proteinaceous channel at the interface between Atp9 ring and Atp6. To prevent unrestricted proton flow dissipating the H^+^-gradient, channel formation is a critical and tightly controlled step during ATP synthase assembly. Here we show that the INA complex (INAC) acts at this decisive step promoting Atp9-ring association with Atp6. INAC binds to newly synthesized mitochondrial-encoded Atp6 and Atp8 in complex with maturation factors. INAC association is retained until the F_1_-portion is built on Atp6/8 and loss of INAC causes accumulation of the free F_1_. An independent complex is formed between INAC and the Atp9 ring. We conclude that INAC maintains assembly intermediates of the F_1_ F_0_-ATP synthase in a primed state for the terminal assembly step–motor module formation.

## Abstract (web summary)

The inner membrane assembly complex (INAC) interacts with components of the F_1_F_0_-ATP synthase but its function remains unclear. Here the authors show that INAC associates with two distinct complexes during F_1_F_0_-ATP synthase formation, which points towards a safeguarding role during proton-conducting channel assembly.

## Introduction

Hydrolysis of ATP to ADP and inorganic phosphate drives biochemical processes in cells. The synthesis of ATP occurs under anaerobic conditions by substrate level phosphorylation during glycolysis, while aerobic conditions allow for oxidative phosphorylation. In eukaryotic cells, the F_1_F_0_-ATP synthase utilizes the proton gradient across the mitochondrial inner membrane, generated by the respiratory chain, to drive ATP formation in the matrix. For this, protons translocate from the intermembrane space across the inner membrane into the matrix through a channel that is formed by Atp6 (subunit *a* in *E*. *coli*) and a ring of Atp9 subunits (subunit *c* in *E*. *coli*). This process converts the electrochemical gradient into a mechanical force eventually catalyzing formation of the anhydride bond in ATP^1–3^.

F_1_F_0_-ATP synthases possess a similar architecture and highly conserved subunits among different eukaryotic species^4^. The *S*. *cerevisiae* enzyme can be divided into two major parts: (i) the matrix-exposed soluble F_1_, which is composed of the catalytic head and a central stalk that translates motor rotation to the catalytic centers; (ii) the membrane-bound F_0_, which contains the peripheral stalk and the membrane-embedded rotor segment. The motor module is built by the ring-like rotor with 10 copies of Atp9 and one molecule of Atp6, which together constitute the proton-translocating membrane channel^5^. The peripheral stalk, formed by Atp4, Atp14, Atp7, and Atp5, interacts with the F_1_ catalytic head and keeps it static, relative to the rotating Atp9 ring^6^. The central stalk, composed of Atp3, Atp15, and Atp16, directly interacts with the Atp9 ring and transmits its rotational movements by elastic power transmission^7^ to the catalytic head, built of three Atp1–Atp2 dimers that each contain a nucleotide-binding pocket^4,8^.

The subunits of the F_1_F_0_-ATP synthase are of dual genetic origin and their stoichiometric assembly into the mature enzyme needs to be coordinated. Most subunits are nuclear-encoded, synthesized as precursors, and imported into the mitochondrion^9^. Only three subunits, Atp6, Atp8, and Atp9, are encoded in the yeast mitochondrial genome and inserted into the inner mitochondrial membrane from the matrix side^10^. Atp6 and Atp8 are encoded on a bi-cistronic mRNA. Translation of this mRNA is regulated by a pre-assembled F_1_ module and Atp22, an Atp6-specific translational activator^11–13^. Upon membrane insertion, Atp6 associates with Atp8 and the Atp10 chaperone, which is required for Atp6 stabilization and subsequent assembly^14–16^. Interestingly, yeast Atp6 is synthesized with a 10 amino acid N-terminal signal sequence that is processed by Atp23, an essential protease of the intermembrane space, required for yeast growth on non-fermentable carbon sources^17–19^. The Atp23/Atp6 interaction, but not the processing of Atp6 per se, is essential for F_1_F_0_-ATP synthase biogenesis as Atp23 also functions as an assembly factor^19^.

Recent studies suggest a stepwise assembly pathway of the F_1_F_0_-ATP synthase that progresses through distinct intermediate modules. It is currently hypothesized that the Atp9 ring associates with a pre-assembled F_1_ module prior to the attachment of an assembly intermediate consisting of Atp6, Atp8, Atp10, and the peripheral stalk^16^. Hence, the proton channel at the interface of the Atp9 ring and Atp6 would form after peripheral stalk and F_1_ are correctly positioned. Although several assembly factors participate in the formation of the individual modules^20–23^, it is unknown how the terminal assembly steps occur and which factors participate in these processes.

**Fig. 1:**
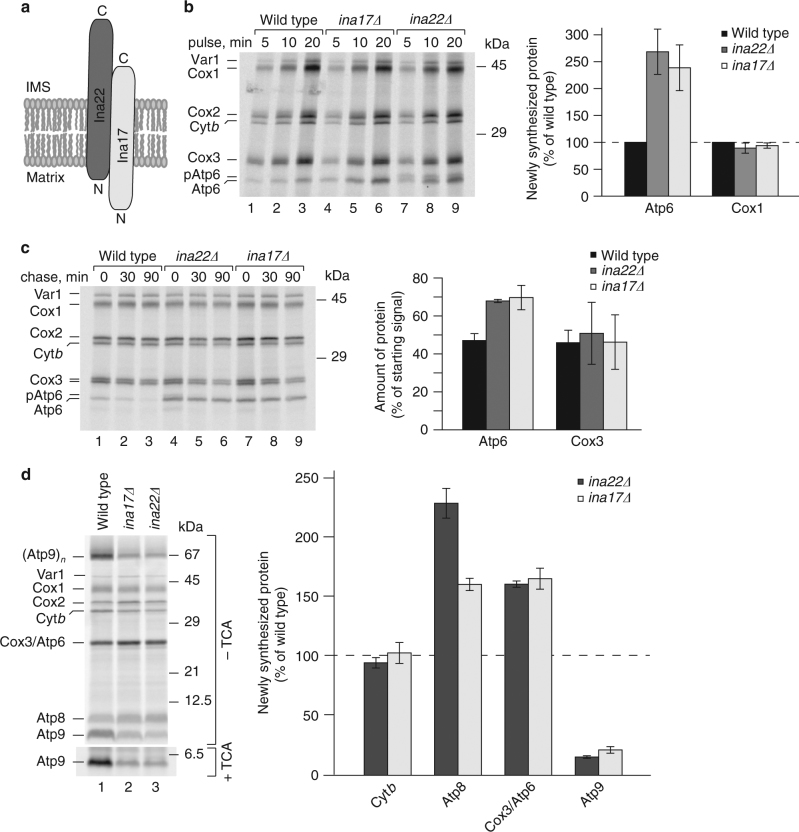
INA complex deficiency affects biogenesis of mitochondrial-encoded F_1_F_0_-ATP synthase subunits. **a** Schematic presentation of the INA complex. IMS, intermembrane space; N, N-terminus, C, C-terminus. **b** Mitochondrial-encoded proteins were radiolabeled in vivo and analyzed by urea SDS-PAGE and digital autoradiography. Atp6 and Cox1 signals obtained after a 20 min pulse were normalized to Cox3 signal and protein amounts in mutant mitochondria were quantified relative to wild type (wild type = 100%), (*n* = 3, ±SEM). **c** Mitochondrial-encoded proteins were radiolabeled in vivo for 10 min. After labeling, samples were chased with an excess of methionine for 30 or 90 min, and analyzed as in **b**. Atp6 and Cox3 signals after 90 min chase were quantified as the percentage of their starting signals (0 min time point), (*n* = 3, ±SEM). **d** Mitochondrial-encoded proteins were radiolabeled in organello for 20 min and analyzed by SDS-PAGE and autoradiography. To quantify Atp9 levels, samples were TCA-precipitated prior to SDS-PAGE and autoradiography. After normalization to Cox1, Atp8, Atp9, Cox3/Atp6, and cytochrome *b* (Cyt*b*) signals in *ina22Δ* and *ina17Δ* mitochondria were quantified as the percentage of wild type (*n* = 3, ±SEM)

The inner membrane assembly complex (INAC) was found to be associated with nuclear-encoded components of the F_1_F_0_-ATP synthase^24^. The complex consists of two single-spanning inner membrane mitochondrial proteins, Ina22 and Ina17 that expose domains into the intermembrane space and interact through coiled-coil motives (Fig. 1a). *ina* mutants display ATP synthase biogenesis defects reflected in dissociation of the F_1_ module. However, the molecular function of INAC remains enigmatic. Here, we report that the INA complex physically associates with two distinct subassemblies of the F_1_F_0_-ATP synthase: the ring of Atp9 subunits as well as with a module consisting of Atp6, Atp8, F_1_, and the peripheral stalk. Our analyses implicate INAC in the terminal step of F_1_F_0_-ATP synthase assembly, the association of Atp9 ring and Atp6. Intriguingly, we find that in the absence of INAC, expression levels of mitochondrial-encoded F_1_F_0_-ATP synthase subunits are readjusted, Atp9 synthesis is reduced, while expression of Atp6 and Atp8 is stimulated. We conclude that INAC association prevents a premature Atp9/Atp6 interaction to safeguard proper F_1_F_0_-ATP synthase assembly and to control the formation of the proton-conducting channel at the interface between Atp9 ring and Atp6.

## Results

### Loss of Ina17 or Ina22 affects translation of Atp6 and Atp8

Ina17 and Ina22 form a complex in the inner membrane of mitochondria exposing domains into the matrix and the intermembrane space^24^ (Fig. 1a). Previously, we have found that a lack of the INA complex affects the biogenesis of the F_1_F_0_-ATP synthase leading to the accumulation of unassembled, free F_1_-subcomplexes. Here, we analyzed the expression of mitochondrial-encoded proteins in *ina*-mutant cells. Interestingly, when mitochondrial translation products were pulse-labeled with [^35^S] methionine, the amount of newly synthesized Atp6 was significantly increased in *ina17*Δ and *ina22*Δ mutants compared with the other translation products (Fig. 1b). To assess whether the increased abundance of Atp6 in the *ina* mutants was due to an increased translation rate or increased stability of Atp6, we carried out pulse-chase analyses. The newly synthesized Atp6 displayed only slightly increased stability in *ina* mutants compared with the wild-type control (Fig. 1c). Accordingly, an increase in translation of Atp6 leads to greater abundance of the newly made protein.

In addition to Atp6, two other subunits of the F_1_F_0_-ATP synthase are mitochondrial-encoded, the small Atp8 and Atp9 proteins. In the inner membrane of mitochondria, Atp9 forms an oligomeric ring consisting of ten protein copies. SDS-PAGE can be used to separate this relatively stable Atp9 oligomer after radiolabeling^25^. To assess Atp9 as well as Atp8 abundance, we pulse-labeled mitochondrial-encoded proteins in organello. Atp9 monomer and oligomer were significantly less abundant in *ina17*Δ and *ina22*Δ-mutant mitochondria (Fig. 1d, upper panel). To quantify Atp9, we dissociated the oligomer by TCA precipitation prior to SDS-PAGE analysis^25^ (Fig. 1d, lower panel). Atp9 was significantly less abundant in *ina* mutants compared with the wild-type control. However, amounts of Atp8 appeared to be increased in *ina* mutants, similar to what was observed for Atp6.

Accordingly, a loss of the INA complex affects the translation of the mitochondrial-encoded F_1_F_0_-ATP synthase subunits, resulting in increased synthesis of Atp6 and Atp8 and decreased Atp9 levels.

### Atp6 processing is affected in *ina22Δ*

**Fig. 2:**
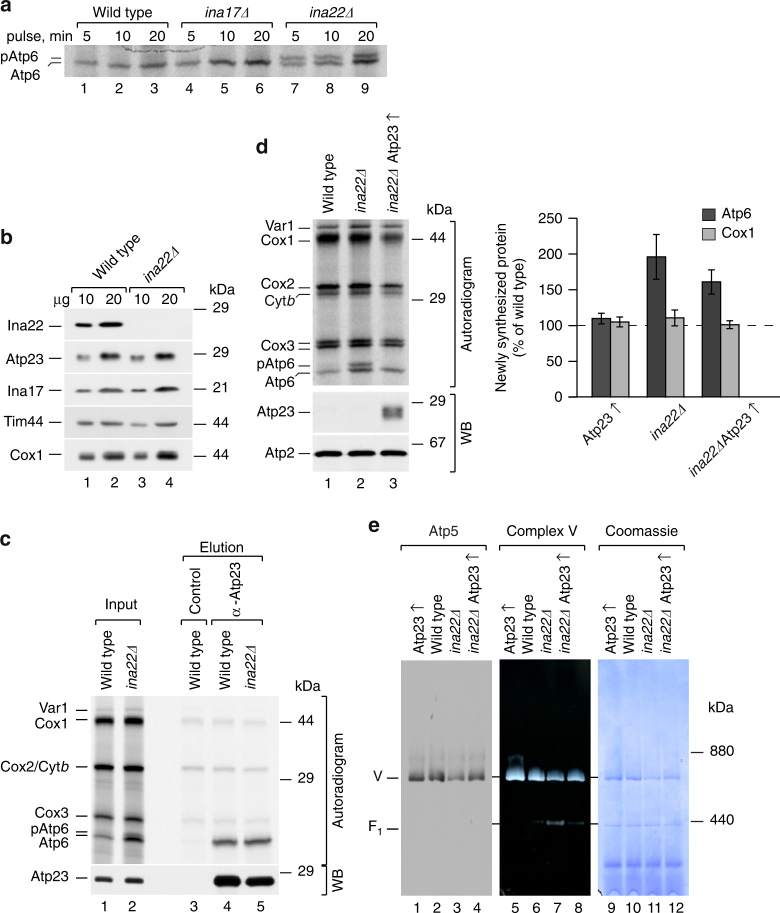
Atp6 processing is affected in *INA22*-mutant mitochondria. **a** Mitochondrial-encoded proteins were radiolabeled in vivo and analyzed by urea SDS-PAGE and digital autoradiography. **b** Wild type and *ina22Δ* mitochondria were solubilized in SDS-sample buffer, subjected to SDS-PAGE and Western blotting with indicated antibodies. **c** Wild type and *ina22Δ* mitochondria were subjected to in organello labeling for 20 min and protein co-immunoprecipitation with control or anti-Atp23 antibodies. Input (1%) and elution (100%) were analyzed by urea SDS-PAGE, digital autoradiography, and western blotting (WB) with anti-Atp23 antibodies. **d** Mitochondrial-encoded proteins were radiolabeled in vivo for 10 min and analyzed by urea SDS-PAGE and digital autoradiography or western blotting (WB) with indicated antibodies (left panel). Quantification was performed as in Figure 1b, *n* = 3, ±SEM. **e** Mitochondria were solubilized with 0.6% dodecyl maltoside (DDM) and analyzed by Blue-Native (BN)-PAGE followed by western blotting with anti-Atp5 antibodies (lanes 1–4), in-gel ATPase activity staining (complex V, lanes 5–8), or Coomassie staining (lanes 9–12)

In yeast mitochondria Atp6 is synthesized as a precursor with a short N-terminal extension, which is processed by Atp23 on the intermembrane space side of the inner mitochondrial membrane^17–19^. To assess Atp6 processing in *ina17*Δ and *ina22*Δ mutants, we radiolabeled mitochondrial translation products and separated precursor and mature forms of Atp6. Although Atp6 processing appeared unaffected in *ina17*Δ, a significant amount of precursor accumulated when Ina22 was lacking (Fig. 2a). To exclude that a lack of Ina22 causes Atp23 deficiency, we analyzed Atp23 protein levels in *ina22*Δ-mutant mitochondria by SDS-PAGE and western blotting. The amount of Atp23 in *ina22*Δ mitochondria was similar to the wild-type control (Fig. 2b) and therefore Atp23 deficiency could be excluded as the reason for Atp6-precursor accumulation. Another possible explanation for the observed Atp6-processing defect in *ina22*Δ could be an impaired interaction between Atp6 and Atp23. Thus, we performed Atp23 immunoprecipitation from wild type and *ina22*Δ-mutant mitochondria in which mitochondrial-encoded proteins were labeled with [^35^S] methionine prior to isolation. Similar amounts of Atp6 were co-immunoprecipitated with Atp23 from mutant and wild-type mitochondria (Fig. 2c), indicating that the interaction between Atp6 and Atp23 was not affected. Considering the increased synthesis of Atp6 in *ina* mutants (Fig. 1b), we speculated that despite unaffected steady-state levels, Atp23 could be rate limiting for Atp6 processing in the context of Ina22 loss. Therefore, we overexpressed Atp23 in the *ina22*Δ strain. First, we verified overexpression by analyzing protein levels in wild type and mutant mitochondria (Supplementary Fig. 1a). As expected, the amount of Atp23 was significantly increased upon overexpression and the protein localized to mitochondria. Next, we assessed whether Atp23 overexpression affected *ina22*Δ growth on non-fermentable carbon sources. In fact, it partially restored the *ina22*Δ growth defect on glycerol (Supplementary Fig. 1b). To directly address whether the observed respiration improvement was due to restored Atp6 processing, we pulse-labeled mitochondrial translation products in wild type and mutant strains. Indeed, Atp23 overexpression rescued Atp6 processing in *ina22*Δ (Fig. 2d, left panel), and partially restored Atp6 translation (Fig. 2d, right panel). As Atp23 was previously shown to play dual roles in Atp6 processing and assembly^19^, we analyzed whether Atp23 overexpression affected F_1_F_0_-ATP synthase assembly in *ina22*Δ. To this end, we analyzed F_1_F_0_-ATP synthase levels in *ina22*Δ and *ina22*ΔAtp23↑ mitochondria by Blue-Native PAGE (BN-PAGE). Western blot analysis showed that Atp23 overexpression restored steady-state levels of F_1_F_0_-ATP synthase in *ina22*Δ (Fig. 2e, lanes 1–4). Moreover, in-gel ATPase activity staining confirmed an obvious reduction of unassembled F_1_-intermediate in *ina22*ΔAtp23↑ compared to *ina22*Δ mitochondria (Fig. 2e, lanes 5–8). Taking together, these data indicate that lack of Ina22 affects the early steps in the biogenesis of Atp6 and thus might also result in impaired Atp6/Atp8 module and peripheral stalk assembly.

### INAC forms a complex with Atp23 and Atp10

**Fig. 3:**
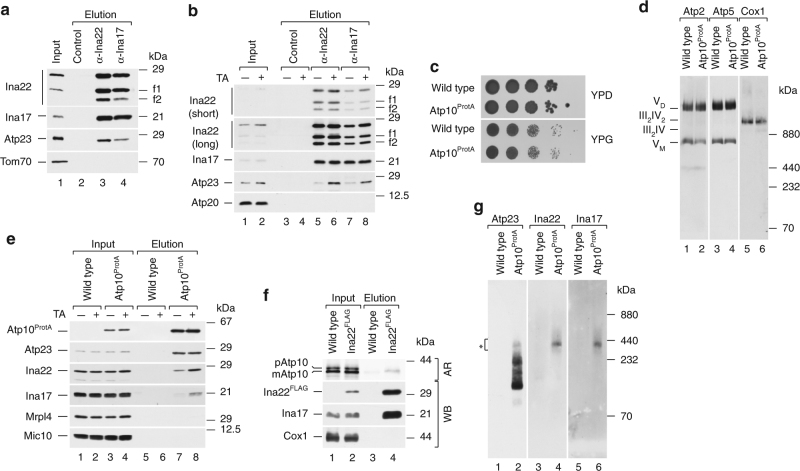
The INA complex associates with Atp23 and Atp10. **a** Proteins from wild-type mitochondria were co-immunoprecipitated with control, anti-Ina22, or anti-Ina17 antibodies. Input and elution fractions were analyzed by SDS-PAGE and western blotting with indicated antibodies. Input = 1% of elution. f1, f2–Ina22 fragments. **b** Proteins from control and chloramphenicol-pretreated wild-type mitochondria were co-immunoprecipitated with control, anti-Ina22, and anti-Ina17 antibodies and analyzed as in **a**. Input = 1% of elution. TA translational activation. f1, f2–Ina22 fragments. **c** Serial dilutions of yeast cells were spotted on YPD or YPG medium. Plates were grown at 37 °C for 5 days. **d** Mitochondria isolated from wild type and Atp10^ProtA^ yeast strains were analyzed by BN-PAGE and western blotting with indicated antibodies. **e** Control and chloramphenicol-pretreated wild type and Atp10^ProtA^ mitochondria were subjected to IgG-affinity chromatography and analyzed as in **a**. Input = 1% of elution. TA translational activation. **f** [^35^S]-labeled Atp10 precursor (p) was imported into wild type or Ina22^FLAG^ mitochondria. Mitochondria were solubilized and subjected to anti-Flag affinity purification. Fractions were analyzed by SDS-PAGE and digital autoradiography (AR, upper panel) or western blotting (WB) with indicated antibodies. Input = 1% of elution. mAtp10, mature Atp10. **g** Protein complexes from Atp10^ProtA^ and wild-type mitochondria were purified via IgG-affinity chromatography, eluted natively via TEV-cleavage, and analyzed by BN-PAGE and western blotting with the indicated antibodies. Chloramphenicol-pretreated mitochondria were used. A star (*) indicates Atp10/Atp23/INAC-containing protein complex

To assess a potential involvement of the INA complex in Atp6/Atp8 module biogenesis, we analyzed whether Ina17 and Ina22 interacted with known Atp6/Atp8 assembly factors, namely Atp23 and Atp10. To immunoprecipitate endogenous INAC, we generated antibodies against the IMS domain of Ina22 and the matrix domain of Ina17. Interestingly, during initial immunoprecipitations two distinct Ina22 fragments (f1 and f2), which represent either products of protein processing or degradation, were co-isolated along with full-length Ina22 from wild type and *ina17*Δ mitochondria (Fig. 3a; Supplementary Fig. 2a). Therefore, we assumed that the observed processing is not Ina17-dependent. Moreover, the fragments were co-immunoprecipitated with Ina17, implying that they still contain a coiled-coil region, required for Ina17–Ina22 interaction^24^. To distinguish between C-terminal and N-terminal processing, we performed Ina22 immunoprecipitation from WT, *ina22*Δ and Ina22^HA^ mitochondria containing a C-terminal 3xHA tag. Only full-length Ina22 could be detected with anti-HA antibodies in both input and elution fractions (Supplementary Fig. 2b, upper panel), whereas decoration with anti-Ina22 antibodies confirmed the presence of two additional fragments in the elution (Supplementary Fig. 2b, lower panel). Accordingly, Ina22 undergoes C-terminal processing. To define the length of the fragments, we generated in vitro a set of C-terminally truncated radiolabeled Ina22 fragments (devoid of a predicted import signal) and compared their electrophoretic mobility to the mobility of immunoprecipitated Ina22 fragments (Supplementary Fig. 2c). We identified that the processing takes place approximately at amino acids 168 and 188 of Ina22. To further investigate a potential function of the processing, we generated yeast mutants expressing truncated Ina22, either 188 (f1) or 168 (f2) amino acids in length, and analyzed for the presence of selected mitochondrial proteins by SDS-PAGE and western blotting. No alteration was observed in the mutant strains (Supplementary Fig. 2d) and their growth on non-fermentable media was not compromised (Supplementary Fig. 2e). Therefore, we conclude that the extreme C terminus of Ina22 does not contribute to its function and that the biological importance of the C-terminal processing is yet to be defined. Moreover, we cannot exclude that the C terminus of Ina22 is unstable and therefore undergoes degradation, generating two stable products, namely f1 and f2.

The observed effect of increased Atp23 levels on *ina22*Δ-mutant cells led us to assess whether INAC interacted with Atp23. Therefore, we immunoprecipitated Ina22 and Ina17 with associated proteins and probed the elution fractions with anti-Atp23 antibodies. Indeed, Atp23 specifically co-purified with INAC (Fig. 3a). We speculated that the INAC–Atp23 association could be facilitated by mitochondrial-encoded proteins. Therefore, we synchronized mitochondrial translations in the wild-type strain by preincubating yeast culture with chloramphenicol for 2 h prior to mitochondria isolation. This treatment results in accumulation of nuclear gene products competent for assembly. Upon release of the translation block, mitochondrial translation is synchronized and assembly of mitochondrial-encoded subunits is stimulated^16,18,26^. Under these conditions, significantly more Atp23 co-immunoprecipitated with INAC (Fig. 3b). We therefore concluded that the INAC–Atp23 interaction is stimulated in the presence of mitochondrial-encoded subunits of F_1_F_0_-ATP synthase.

Atp10 was previously shown to associate with newly translated Atp6, to stabilize it and to facilitate its assembly^15,16^. To address a possible Atp10–INAC interaction, we genomically fused protein A from *S*. *aureus* to the C terminus of Atp10. The fusion protein remained functional, as Atp10^ProtA^-expressing cells displayed wild type-like growth on non-fermentable carbon sources (Fig. 3c). Moreover, a BN-PAGE analysis of F_1_F_0_-ATP synthase complexes in wild type and Atp10^ProtA^ mitochondria revealed their similar steady-state amounts (Fig. 3d). To assess an INAC–Atp10 interaction and if it was stimulated by mitochondrial translation products, we isolated Atp10^ProtA^-associated protein complexes from control and chloramphenicol-treated mitochondria. INAC co-isolated with Atp10^ProtA^ and a significant increase in co-purification was observed upon mitochondrial translational stimulation (Fig. 3e). Ina22^FLAG^ isolation after import of radiolabeled Atp10 confirmed the Ina22–Atp10 interaction (Fig. 3f). Surprisingly, in addition to INAC, Atp23 co-isolated with Atp10^ProtA^, in contrast to previous findings^16^ (Fig. 3e). However, the amount of Atp23 that was co-isolated with Atp10 did not change upon mitochondrial translational upregulation, implying that no free Atp23 pool exists in mitochondria and all Atp23 remains associated with complex V assembly intermediates. We wondered whether a protein complex containing Atp23, Atp10, and INA proteins could be isolated. Therefore, Atp10^ProtA^-associated proteins from chloramphenicol-treated mitochondria were purified, natively eluted via TEV-protease cleavage and analyzed by BN-PAGE. Strikingly, an Atp23, Ina22, and Ina17-containing complex of 400 kDa was detected (Fig. 3g). In summary, we conclude that INAC physically interacts with Atp23 and Atp10 in a common protein complex.

### Peripheral stalk and F_1_ assemble to an Atp6/Atp8 module

**Fig. 4:**
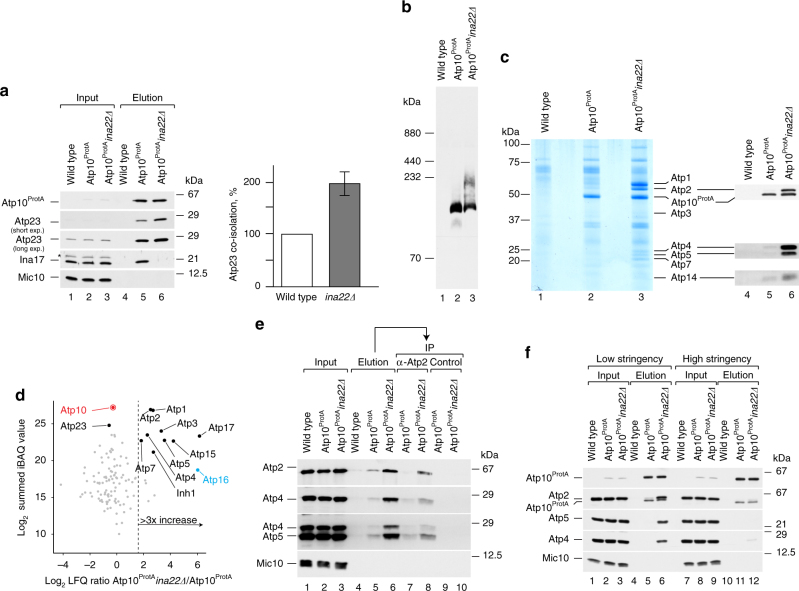
Atp10 accumulates with Atp23, peripheral stalk and F_1_ module in *ina22Δ*. **a** Atp10^ProtA^-containing protein complexes were purified via IgG-affinity chromatography from Atp10^ProtA^, Atp10^ProtA^ *ina22Δ*, and wild-type mitochondria. Input and elution fractions were analyzed by SDS-PAGE and western blotting with indicated antibodies (left panel). The amount of Atp23 isolated from Atp10^ProtA^ mitochondria was set as 100% and Atp23 co-isolated from Atp10^ProtA^ *ina22Δ* mitochondria was quantified (right panel). Signals were normalized to the amounts of isolated Atp10^ProtA^. Input = 1% of elution. (*n* = 3, ±SEM). The asterisk (*) indicates a cross-reacting band. **b** The experiment was performed as in **a**, bound complexes were eluted natively and analyzed by BN-PAGE and western blotting with anti-Atp23 antibodies. **c** Wild type, Atp10^ProtA^, and Atp10^ProtA^ *ina22Δ* mitochondria were subjected to IgG-affinity chromatography. Ninety percent of the elution fractions was analyzed by SDS-PAGE and colloidal Coomassie staining (lanes 1–3). Remaining fractions were analyzed by SDS-PAGE and western blotting with indicated antibodies (lanes 4–6). **d** Atp10^ProtA^-containing protein complexes were purified via IgG-affinity chromatography from Atp10^ProtA^ and Atp10^ProtA^ *ina22Δ* mitochondria. Eluates were analyzed by BN-PAGE followed by LC-MS and label-free quantification using the MaxQuant LFQ and the iBAQ algorithm. The scatter plot shows for each protein the logarithmic (log_2_) ratio of LFQ intensities for Atp10^ProtA^ *ina22Δ* over Atp10^ProtA^ plotted against the summed iBAQ intensity of Atp10^ProtA^ *ina22Δ* and Atp10^ProtA^. The dashed line indicates a non-logarithmic ratio of three. Red, bait protein; blue, Atp16 was only identified in Atp10^ProtA^ *ina22Δ* and the ratio was set to six. **e** Wild type, Atp10^ProtA^, and Atp10^ProtA^ *ina22Δ* mitochondria were subjected to IgG-affinity chromatography. Protein complexes were eluted natively via TEV-cleavage. Elution fractions were subjected to immunoprecipitation (IP) with either anti-Atp2 antibodies or a control pre-immune serum. 1% of the input fraction and entire elution fraction were analyzed by SDS-PAGE and western blotting with indicated antibodies. **f** Wild type, Atp10^ProtA^, and Atp10^ProtA^ *ina22Δ* mitochondria were solubilized in a high- or low-stringency buffer (see “Experimental procedures”) and subjected to IgG-affinity chromatography. Input and elution fractions were analyzed by SDS-PAGE and western blotting with indicated antibodies. Input = 1% of elution

The peripheral stalk, Atp6, Atp8, and Atp10 have been proposed to form a pre-assembled module devoid of Atp23^16^. However, our analyses show that Atp10 associates with Atp23 in multiple protein complexes with and without INA proteins (Fig. 3g). If INAC associates with the Atp6/Atp8 module to facilitate peripheral stalk assembly, we wondered whether loss of Ina22 would stall the assembly and result in accumulation of Atp10- and Atp23-containing intermediates. Therefore, Atp10^ProtA^ complexes were isolated from wild type and *ina22*Δ mitochondria. Not unexpectedly, Atp23 accumulated in a complex with Atp10 in *ina22*Δ, strongly suggesting that they are part of the same assembly intermediate (Fig. 4a). Moreover, a BN-PAGE analysis of the elution fractions showed an accumulation of Atp10- and Atp23-containing complex in *ina22*Δ mitochondria (Fig. 4b). We assessed the composition of the accumulated complex by preparatory scale purification. SDS-PAGE analysis of Atp10^ProtA^-isolated protein complexes followed by Coomassie staining revealed a significantly different protein pattern in the *ina22*Δ sample compared to the wild-type situation (Fig. 4c, lanes 2–3). Western blot analysis unexpectedly revealed an accumulation of nuclear-encoded F_1_ and peripheral stalk subunits in complex with Atp10 upon *INA22* deletion (Fig. 4c, lanes 4–6). BN-PAGE analyses of the elution fractions showed extensive accumulation of Atp4-containing protein complexes (peripheral stalk), Atp1–Atp2 dimer, fully assembled F_1_ module and a complex of intermediate size upon *INA22* deletion (Supplementary Fig. 3a). We concluded that Atp2-containing complexes represented fully and partially assembled F_1_. To additionally verify this, we analyzed the composition of the Atp10^ProtA^-containing protein complexes isolated from WT and *ina22*Δ mitochondria by mass spectrometry. In agreement with the western blot analysis (Fig. 4c), we confirmed a significant accumulation of the F_1_ and peripheral stalk subunits with Atp10 upon *INA22* deletion (Fig. 4d; Supplementary Data 1). Accordingly, the Atp6/Atp8 module would serve as an assembly platform for the F_1_-portion and the peripheral stalk. However, since the Atp2- and Atp4-containing complexes did not co-migrate on BN-PAGE (Supplementary Fig. 3a), we considered that either two distinct Atp10-containing assembly intermediates exist, associated with either peripheral stalk or F_1_, or that a single intermediate containing peripheral stalk and F_1_ dissociated to smaller subcomplexes under BN-PAGE conditions. To distinguish between these two scenarios, we performed a two-step affinity purification of these complexes. First, Atp10^ProtA^-containing assembly intermediates were natively isolated. Second, the obtained elution fractions were used for immunoprecipitation with either anti-Atp2 antibodies or a control pre-immune serum. The western blot analyses of all elution fractions clearly showed that Atp2 remains associated with Atp4 and Atp5, two constituents of the peripheral stalk, within a single Atp10-associated protein complex (Fig. 4e). This finding was surprising as association of the Atp6/Atp8 module with F_1_ or Atp5 was not detected previously^16^. We noticed that buffer conditions were the main difference between this and our analysis. Rak et al.^16^ used relatively high salt conditions and high pH. To check whether the identified complex was unstable under these conditions, we isolated Atp10^ProtA^-containing protein complexes from mitochondria, solubilized in two different buffers (low stringent and high stringent). As expected, the Atp10 interaction with F_1_ and the peripheral stalk was salt-sensitive (Fig. 4f), which might explain why this intermediate was not detected before.

Next, we wondered whether *INA22* deletion affected the interactome of Atp23 similar to what was found for Atp10 (Fig. 4d). To address this, we performed Atp23 immunoprecipitations from wild type and *ina22*Δ mitochondria, and analyzed the elution fractions by quantitative mass spectrometry (Supplementary Data 2). As expected, in the absence of Ina22, only slightly increased amounts of nuclear-encoded ATP synthase subunits were recovered with Atp23. This finding supports the idea that in mitochondria most or all of Atp23 is associated with complex V assembly intermediates.

In summary, a large assembly intermediate accumulates upon loss of INAC, which contains the Atp6/Atp8 module associated with Atp23 and Atp10, the peripheral stalk, and F_1_-portion. This intermediate is transient in wild-type mitochondria, dissociates upon BN-PAGE analysis and under high ionic force.

### Ina22 is required for Atp9-ring assembly

**Fig. 5:**
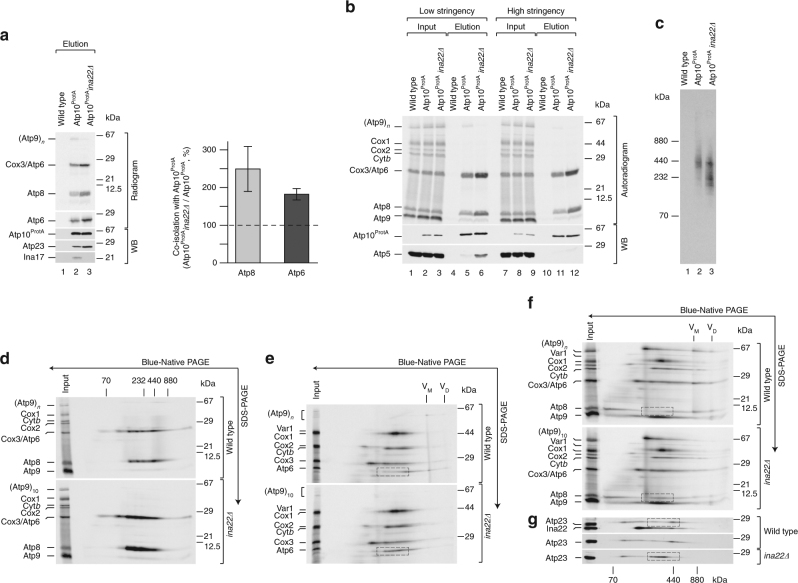
Atp10^ProtA^ accumulates with Atp6/Atp8-containing assembly intermediate in *ina22Δ*. **a** Chloramphenicol-pretreated wild type, Atp10^ProtA^, and Atp10^ProtA^ *ina22Δ* mitochondria were subjected to in organello labeling for 20 min and IgG-affinity chromatography. Input and elution fractions were analyzed by SDS-PAGE (for Atp8 quantification) or urea SDS-PAGE (for Atp6 quantification), followed by digital autoradiography and western blotting (WB). Amounts of Atp6 and Atp8, isolated from Atp10^ProtA^ mitochondria, were set to 100%. Signals in the elution fraction were normalized to the amount of isolated Atp10^ProtA^, (*n* = 3, ±SEM). **b** Chloramphenicol-pretreated wild type, Atp10^ProtA^, and Atp10^ProtA^ *ina22Δ* mitochondria were subjected to in organello labeling for 20 min and IgG-affinity chromatography under low-stringency conditions and high-stringency conditions. Input and elution fractions were analyzed by SDS-PAGE, digital autoradiography, and western blotting (WB). Input = 1% of elution. **c** Chloramphenicol-pretreated wild type, Atp10^ProtA^ and Atp10^ProtA^ *ina22Δ* mitochondria were subjected to in organello labeling for 20 min and IgG-affinity chromatography. Complexes were eluted natively and elution fractions were analyzed by BN-PAGE and digital autoradiography. **d** Part of the elution fraction of the experiment presented in **b**, was analyzed by BN-PAGE, second dimension SDS-PAGE and digital autoradiography. **e**, **f** Chloramphenicol-pretreated wild type and *ina22Δ* mitochondria were subjected to in organello labeling for 15 min. Protein complexes were analyzed by BN-PAGE and second dimension urea SDS-PAGE (**e**) or SDS-PAGE (**f**) followed by digital autoradiography. Dashed boxes show Atp6/Atp8-containing assembly intermediates. **g** Chloramphenicol-pretreated wild type and *ina22Δ* mitochondria were analyzed by BN-PAGE, second dimension SDS-PAGE, and western blotting with the indicated antibodies. Dashed boxes show Atp23-containing complexes

To analyze whether Atp6 and Atp8 accumulate in a complex with Atp10, Atp23, the peripheral stalk, and the F_1_-portion in *ina22*Δ mitochondria, we radiolabeled mitochondrial-encoded proteins in chloramphenicol-treated wild type, Atp10^ProtA^, and Atp10^ProtA^ *ina22*Δ mitochondria, and performed isolation of Atp10^ProtA^-containing protein complexes. Compared to wild type, twice as much Atp6 and Atp8 co-isolated with Atp10 in *ina22*Δ mitochondria (Fig. 5a). This increase mirrors the observed enhanced Atp10–Atp23 interaction (Fig. 4a). Taking our previous findings into consideration (Fig. 4f), we wondered whether Atp6 and Atp8 remained associated with Atp10 under high-stringency conditions or dissociate from Atp10-containing protein complexes, similar to the F_1_ module and the peripheral stalk. To this end, we radiolabeled mitochondrial-encoded proteins in wild type, Atp10^ProtA^, and Atp10^ProtA^ *ina22*Δ mitochondria, and performed isolation of Atp10^ProtA^-containing protein complexes under low-stringency and high-stringency conditions. In contrast to the F_1_ module and the peripheral stalk, Atp6 and Atp8 remained attached to Atp10 even under high-stringency conditions (Fig. 5b), in agreement with previously published data^16^.

On the basis of these findings, we aimed to analyze accumulated Atp6 and Atp8-containing protein complexes in *ina22*Δ mitochondria by BN-PAGE and autoradiography. One prominent assembly intermediate of 400 kDa co-isolated from wild type and *ina22*Δ mitochondria, whereas several additional smaller-size assembly intermediates accumulated in *ina22*Δ (Fig. 5c). Second dimension SDS-PAGE electrophoresis confirmed that these complexes in fact contained Atp6 and Atp8 (Fig. 5d).

To understand the molecular bases for the observed assembly stalling in the absence of INAC, mitochondrial proteins were radiolabeled in wild type and *ina22*Δ mitochondria. Assembly intermediates were analyzed by first dimension BN-PAGE and second dimension SDS-PAGE. For better separation of Atp6 from Cox3 and Atp8 from Atp9, two different gel systems were applied (Fig. 5e, f). The analyses showed that loss of Ina22 led to accumulation of Atp6 and Atp8 in smaller assembly intermediates. Atp23 accumulated together with Atp6 and Atp8 in a common complex (Fig. 5g). Remarkably, after a 15 min pulse sufficient amounts of completely assembled complex V monomer and dimer could be detected in the wild-type mitochondria, whereas in *ina22*Δ neither Atp6, Atp8, nor Atp9 ring were assembled into F_1_F_0_-ATP synthase monomer (Fig. 5e, f). Therefore, we concluded that assembly of F_1_F_0_-ATP synthase is stalled right before the last assembly step, namely Atp9-ring association.

### INAC mediates the last F_1_F_0_-ATP synthase assembly step

**Fig. 6:**
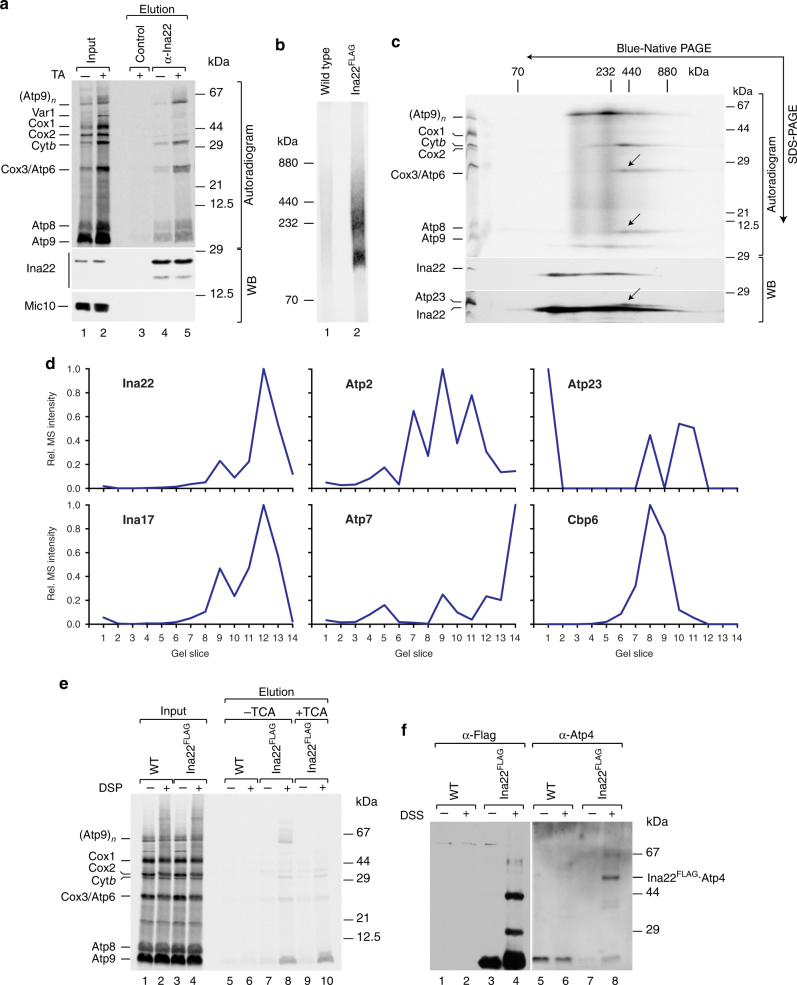
Ina22 interacts with mitochondrial-encoded F_1_F_0_-ATP synthase subunits. **a** Mitochondrial-encoded proteins in control and chloramphenicol-pretreated wild-type mitochondria were labeled in organello for 20 min. Proteins were co-immunoprecipitated with control or anti-Ina22 antibodies and samples were analyzed by SDS-PAGE, digital autoradiography, and western blotting (WB) with the indicated antibodies. Input = 1% of elution. TA, translational activation. **b** Chloramphenicol-pretreated wild type and Ina22^FLAG^ mitochondria were subjected to in organello labeling, anti-FLAG affinity chromatography, and further analysis by BN-PAGE and digital autoradiography. **c** Part of the Ina22^FLAG^ elution fraction of the experiment presented in **b** was subjected to BN-PAGE, second dimension SDS-PAGE, digital autoradiography, and western blotting (WB). Arrows indicate Atp6/Atp8 module, co-isolated with Ina22^FLAG^. **d** Ina22^FLAG^-containing protein complexes were immunopurified from Ina22^FLAG^ mitochondria using anti-FLAG antibody. Eluates were analyzed by BN-PAGE and LC-MS followed by the establishment of protein abundance profiles. For each protein, summed MS intensities measured in each gel slice were calculated, the maximum intensity was set to 1, and normalized values were plotted across the 14 gel slices. **e** Wild type and Ina22^FLAG^ mitochondria were subjected to chemical crosslinking after radiolabeling of mitochondrial-encoded proteins and Ina22^FLAG^ was purified under denaturing conditions. Input and elution fractions were analyzed by SDS-PAGE and digital autoradiography. Elution fractions were split and either left untreated (lanes 5–8) or precipitated with 12.5% TCA to disrupt Atp9 oligomer (lanes 9–10). **f** Wild type and Ina22^FLAG^ mitochondria were subjected to chemical crosslinking and Ina22^FLAG^ was purified under denaturing conditions. Elution fractions were analyzed by SDS-PAGE and western blotting with anti-FLAG or anti-Atp4 antibodies

Our analyses show that the last F_1_F_0_-ATP synthase assembly step, the connection of Atp6/Atp8-containing assembly intermediates with the Atp9 ring, is impaired by a lack of INAC. Thus, we asked whether the INA complex mediates this step. To directly address this, we carried out Ina22 immunoprecipitation after radiolabeling of mitochondrial translation products in control and chloramphenicol-treated mitochondria. Ina22 was found associated with Atp6, Atp8, and Atp9 (Fig. 6a). As only minor amounts of these proteins were co-isolated with Ina22 from a non-treated sample and their interaction was significantly increased upon translational upregulation, we conclude that this interaction is transient in nature. Moreover, we noticed that Ina22 associated with newly translated cytochrome *b* in a similar fashion (Fig. 6a). Taking our previous findings into consideration, which showed that the INA complex associates with the cytochrome *b* biogenesis factors Cbp3, Cbp6, and Cbp4 but has no role in complex III biogenesis^24^, we asked whether the Cbp proteins are involved in assembly of the F_1_F_0_-ATP synthase. To address this, we tagged Cbp3, Cbp4, and Cbp6 with a C-terminal Protein A tag and isolated protein complexes by IgG-affinity chromatography. Clearly, Cbp3, Cbp4, and Cbp6 associated with the INA complex, but not with the structural subunits of the F_1_F_0_-ATP synthase (Supplementary Fig. 4a). Moreover, isolation of Cbp3^ProtA^-containing complexes after radiolabeling of mitochondrial-encoded proteins confirmed that Cbp3^ProtA^ interacts with newly synthesized cytochrome *b*, but not with Atp6 or Atp9 (Supplementary Fig. 4b). BN-PAGE and second dimension SDS-PAGE analyses of these complexes showed that the INA complex components associated with Cbp3 and Cbp4 and newly translated cytochrome *b* (Supplementary Fig. 4c, d). Despite this association, the INA complex was found dispensable for complex III biogenesis^24^. To address a role of the Cbp proteins in the biogenesis of the F_1_F_0_-ATP synthase, we assessed the levels and activity of mature F_1_F_0_-ATP synthase by BN-PAGE analyses in wild type and *cbp*-mutant mitochondria. To this end no differences were observed between mutant and wild type indicating that Cbp3, Cbp4, and Cbp6 are expendable for biogenesis of complex V (Supplementary Fig. 4e).

We asked whether Ina22 was present in two distinct complexes, one containing the Atp9 ring and the other containing Atp6 and Atp8. For this, we tagged Ina22 with a C-terminal Flag-tag, verified that it did not affect its association with newly translated F_1_F_0_-ATP synthase components (Supplementary Fig. 5), and performed native isolation of Ina22^FLAG^-containing protein complexes after radiolabeling of mitochondrial translation products. BN-PAGE analysis of the elution fractions showed that Ina22 associated with several assembly intermediates (Fig. 6b) and further second dimension SDS-PAGE analysis confirmed one of them to contain the Atp9 ring, and another one to contain Atp6, Atp8, and Atp23 (Fig. 6c, arrows). A mass spectrometric analysis of the Ina22^FLAG^-isolated protein complexes separated by BN-PAGE supported the protein distribution observed by western blotting in the second dimension (Fig. 6d; Supplementary Data 3).

To address whether the interaction of Ina22 with newly translated F_1_F_0_-ATP synthase components was direct, we performed chemical crosslinking after radiolabeling of mitochondrial-encoded proteins in wild type and Ina22^FLAG^ mitochondria, and isolated Ina22^FLAG^ under denaturing conditions. Strikingly, the Atp9 ring as well as monomeric Atp9 could be crosslinked to Ina22^FLAG^, confirming their direct interaction (Fig. 6e). As expected, we also found crosslinks of Atp6 and cytochrome *b* with Ina22. In addition, we obtained Ina22 crosslink to Atp4, suggesting that Ina22–Atp6/Atp8 interaction involves the peripheral stalk (Fig. 6f).

**Fig. 7:**
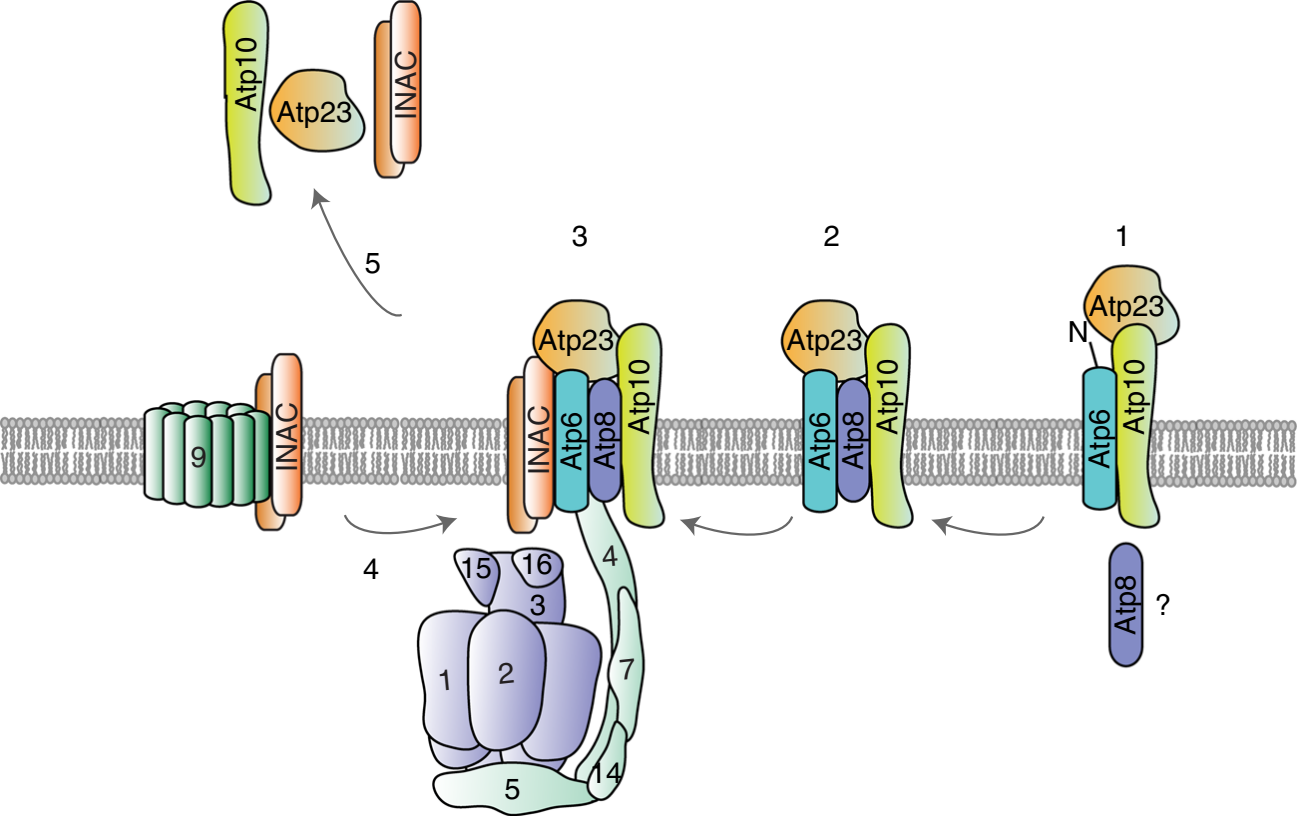
A model of F_1_F_0_-ATP synthase assembly. After insertion into the inner mitochondrial membrane, Atp6 associates with Atp10 and Atp23 and undergoes N-terminal processing (state 1). Moreover, Atp6 interacts with Atp8 to form an early assembly intermediate, which consists of Atp6, Atp8, Atp10, and Atp23–Atp6/8 module (state 2). Presumably, the Atp6/8 module serves as an assembly platform for the peripheral stalk and F_1_ (state 3). Moreover, the INA complex associates with this later assembly intermediate and, most likely, keeps it in a primed state for Atp9 oligomer association. In addition, the INA complex interacts with the Atp9 oligomer and facilitates formation of the proton-conducting channel in the last step of F_1_F_0_-ATP synthase assembly (step 4). After the last assembly step, assembly factors are released and recycled (step 5)

We conclude that the INA complex physically associates with two distinct F_1_F_0_-ATP synthase assembly intermediates, the Atp9 ring and the module consisting of the F_1_-portion together with the peripheral stalk, Atp6 and Atp8, prior to their association. Accordingly, INAC mediates the terminal assembly step, the association of the Atp9 ring to form the proton-conducting channel with Atp6 (Fig. 7).

## Discussion

The biogenesis of the F_1_F_0_-ATP synthase requires a stoichiometric association of subunits expressed in mitochondria and the cytoplasm. Compared to the biogenesis of respiratory chain complexes, little molecular detail is known on the assembly of the F_1_F_0_-ATP synthase and only a relatively small number of assembly factors promoting this process have been defined^27^. In bacteria, the insertase YidC facilitates membrane insertion of the c-subunit^28^. Atp1/Unc1 has been found to subsequently support c-ring (homolog to subunit 9) assembly^29^. In yeast, the YidC-related insertase Oxa1 acts as a general insertase for mitochondrial-encoded proteins^30^. So far, no Atp1/Unc1-related assembly factor specifically assisting in Atp9-ring formation has been identified in yeast. Moreover, proteins that act at the stage of liasing the Atp9-ring with Atp6/Atp8 have not been defined. Our analyses now provide molecular insights into the function of the INA complex, which appears to have no bacterial homolog, in F_1_F_0_-ATP synthase assembly and the critical step of proton channel formation.

Here we find that the mitochondrial-encoded Atp6 and Atp8 proteins associate with the INA complex. This association between INAC and newly synthesized mitochondrial-encoded subunits of the F_1_F_0_-ATP synthase was not as apparent in our initial analyses as only small amounts of these proteins were co-purified with Ina17^ProtA^ and Ina22 had not been tested in this regard^24^. At this point, we cannot exclude that the tag on Ina17 affected the co-purification of mitochondrial-encoded subunits to some extent. Therefore, the use of antibodies against the authentic proteins appears to provide the most reliable results regarding association of mitochondrial-encoded subunits. In addition to an association with subunits of the F_1_F_0_-ATP synthase, INAC is found together with early assembly intermediates of cytochrome *b*. The physiological significance of this interaction remains elusive. The INA complex subunits appear to be neither essential for complex III biogenesis nor did we find a defect in complex V biogenesis upon loss of Cbp proteins.

Our analyses show that the INA complex is required for Atp6 maturation by Atp23. As overexpression of Atp23 suppresses the Atp6 processing defect of *ina22Δ* mutant, we conclude that the amount of Atp23 for processing of Atp6 becomes limited when Ina22 is lacking. However, it is unclear at which time point during assembly of Atp6 the processing event actually occurs and therefore it is uncertain how early the INA complex engages with Atp6. Besides being a protease for Atp6 maturation, Atp23 has been found to suppress *atp10*-mutant cells and to act as an Atp6-specific chaperone^19^. Interestingly, our data show now that Ina22, Ina17, Atp10, and Atp23 form a complex together. In a detailed analysis on F_1_F_0_-ATP synthase complex formation, Rak et al.^16^ identified an interaction between Atp6, Atp8, and Atp10. However, Atp23 was not found associated. Why has complex formation between these proteins not been detected? In the course of our analyses, we found that C-terminal tagging of Atp23 affects protein stability and its association with the Atp6/Atp8 module (Supplementary Fig. 3b). Thus, the use of a tagged version of Atp23 could have prevented detection of the above-mentioned complex. It is interesting to note that Atp10 and Atp23 remain associated with Atp6 and Atp8 until the F_1_-portion and the peripheral stalk have engaged with these motor subunits. This unexpected finding shows that building the F_1_-portion on the Atp9-ring is not a mandatory assembly step for the F_1_F_0_-ATP synthase. As the Atp6/Atp8, F_1_ and the peripheral stalk complex are sensitive towards higher salt concentrations, we speculate that ionic interactions have an important role for this subassembly. This fact might explain as to why this assembly state has so far not been observed. A lack of INAC causes accumulation of this biogenesis intermediate indicating a role of Ina22 and Ina17 downstream of F_1_ and peripheral stalk binding to the Atp6/Atp8 motor module.

Ina22 crosslinks to Atp4, a subunit of the peripheral stalk, as well as to the Atp9-ring. This observation places Ina22 at two critical sites for F_1_F_0_-ATP synthase assembly and function. As Atp4 is positioned on Atp6, our results place INAC in the inner membrane at the lateral side of the Atp6/Atp8 motor module. Considering that a lack of INAC affects association of the F_1_-portion with the Atp9-ring, we conclude that INAC is involved in liaising the two motor modules of the F_1_F_0_-ATP synthase. Atp6 and Atp9 ring form a direct protein-protein interface that builds the proton-conducting channel. Our results are in agreement with the idea that both of these interfaces display hydrophilic regions that are unlikely to be easily exposed to a lipid environment. In fact, structural analyses of the F_1_F_0_-ATP synthase display charged surfaces on Atp9 and Atp6^31^. We suggest that the INA complex interacts with the transmembrane segments of Atp6 and Atp9 until the terminal assembly step can occur to form the proton-conducting interface.

## Methods

### Yeast strains and growth conditions

All yeast strains used in this study are listed in Supplementary Table 1 and are derivatives of the BY4741 strain^32^. The wild-type BY4741 strain as well as *ina22*Δ and *ina17*Δ were obtained from the Euroscarf collection. All strains were generated by homologous recombination of PCR-derived cassettes amplified from plasmids pYM10, pYM2, pFA6a-HISMX6, pYM-N15^33^, or pUG72^34^ with oligonucleotides provided in Supplementary Table 2. All strains were grown on YPD (1% yeast extract, 2% peptone, 2% glucose), YPGal (1% yeast extract, 2% peptone, 2% galactose), or YPG (1% yeast extract, 2% peptone, 3% glycerol) at 30 °C. Mitochondria were isolated from yeast grown on YPG or YPGal as described previously^35^. In brief, yeast were pre-cultured in 5 mL of YPD media and then sequentially in 100 and 200 mL of either YPG or YPGal. The last pre-culture was used to inoculate the main culture. Flasks were incubated overnight at 30 °C with shaking (190 rpm) until OD600 reached 1.5–2.0. Cells were pelleted, washed with water, and resuspended in 10 mM DTT, 100 mM Tris/HCl pH 9.4 followed by a 30 min incubation at 30 **°**C with shaking. The cells were again collected by centrifugation, washed with 1.2 M sorbitol, and resuspended in zymolyase buffer (1.2 M sorbitol, 20 mM potassium phosphate pH 7.4, 4 mg of zymolyase per 1 g of initial yeast pellet) followed by 1 h incubation at 30 **°**C with shaking. Treated cells were washed once with zymolyase buffer without the enzyme, resuspended in homogenization buffer (0.6 M sorbitol, 10 mM Tris/HCl pH 7.4, 1 mM EDTA, 0.2% (w/v) fatty acid-free BSA, 1 mM PMSF) and homogenized by 15 strokes at 800 rpm in Potter S glass Teflon homogenizer. The mitochondrial fraction was obtained by differential centrifugation. The mitochondrial pellet was washed once with 250 mM sucrose, 10 mM MOPS-KOH pH 7.2, 1 mM EDTA, 2 mM PMSF, and resuspended in this buffer to final concentration 10 mg mL^−1^. Isolated mitochondria were stored in single-use aliquots at −80 °C.

### Radiolabeling of mitochondrial translation products

In vivo labeling of mitochondrial translation products in cells grown on rich medium containing 3% glycerol was performed as previously described with modifications^36^. In brief, yeast cells were grown overnight in YPG medium at 30 **°**C with agitation (200 rpm). Yeast cultures were diluted to OD600 = 0.4 and grown in YPG until OD600 = 1. The amount of cells corresponding to 0.3 OD600 was taken, cells were pelleted by centrifugation and washed once with 50 mM phosphate buffer pH 7.2, 2% galactose, resuspended in this buffer, and incubated at 30 **°**C for 10 min, 600 rpm. Next, 150 μg mL^−1^ cycloheximide was added and cells were incubated for five more minutes. To start labeling of mitochondrial-encoded proteins, 20 mCi of [^35^S]Met were added and labeling was performed for the required time period. Labeling was stopped by addition of 10 mM unlabeled methionine. To address protein stability, samples were additionally incubated at 30 **°**C. Cells were spun down, washed once with water and yeast whole-cell lysates were prepared. Proteins were precipitated with TCA and analyzed by SDS-PAGE or urea SDS-PAGE, western blotting, and digital autoradiography. In organello labeling of mitochondrial proteins was performed in presence of 900 mM sorbitol, 225 mM KCl, 22.5 mM potassium phosphate buffer pH 7.4, 30 mM Tris/HCl pH 7.4, 4.5 mg mL^−1^ bovine serum albumin, 6 mM ATP, 0.75 mM GTP, 9 mM α-ketoglutarate, 10 mM creatine phosphate, 0.15 mM methionine-free amino acid mix, 150 μg mL^−1^ cycloheximide, 19 mM MgSO_4_, and 130 µg mL^−1^ creatine kinase. In brief, samples were prewarmed to 30 **°**C and the reaction was started by addition of 20 mCi of [^35^S] methionine. The reaction was chased with 10 mM unlabeled methionine when needed. Mitochondria were reisolated and washed in SEM buffer (250 mM saccharose, 1 mM EDTA, 10 mM MOPS). Samples were either used for further affinity purification or directly analyzed. For analysis, samples were separated by SDS-PAGE or urea-PAGE, proteins were transferred to a PVDF membrane and the membrane was exposed to a storage phosphor screen (GE Healthcare, USA). Signals were digitalized using a Storm820 scanner (GE Healthcare, USA). Radioactive signals were quantified using the ImageG software.

### Affinity purification of protein complexes

For IgG-affinity chromatography of Atp10^ProtA^-containing protein complexes, CNBr-sepharose (GE Healthcare, USA) was coupled to human IgG (Sigma Aldrich) according to the manufacturer’s instructions. For isolation of FLAG-conjugated bait, anti-FLAG M2 Affinity Gel (Sigma Aldrich, USA) was used. For immunoprecipitation of Ina22, Ina17, Atp2, and Atp23, the corresponding rabbit antiserum was coupled to Protein A sepharose (GE Healthcare, USA) using dimethyl pimelimidate. In brief, mitochondria were solubilized in a buffer containing 1% digitonin, 160 mM NaCl, 20 mM Tris-HCl, pH 7.5, 10% glycerol, and 2 mM phenylmethylsulfonyl fluoride (PMSF) for 30 min at 4 °C. For the comparison of low-stringent and high-stringent solubilization conditions, mitochondria were solubilized in a high-stringent buffer containing 1% digitonin, 400 mM NaCl, 10 mM Tris-HCl pH 9.0, 2 mM PMSF (as previously described^16^). Non-solubilized material was removed by centrifugation at 16,000×*g* for 10 min and the supernatant was mixed with beads. After 1.5 h binding at 4 °C, the beads were washed with 0.3% digitonin buffer containing 160 mM NaCl, 20 mM Tris-HCl, pH 7.5, 10% glycerol (or 400 mM NaCl, 10 mM Tris-HCl, pH 9.0 for high-stringent conditions). Bound material was eluted with 0.1 M glycine pH 2.8, FLAG-peptide, or with 0.4 mg mL^−1^ tobacco etch virus protease (AcTEV; Thermo Fisher Scientific, USA) overnight at 4 °C.

For isolation of Ina22^FLAG^ under denaturing conditions, mitochondria were solubilized in a buffer containing 1% SDS, 160 mM NaCl, 20 mM Tris-HCl, pH 7.5, 10% glycerol, and 2 mM PMSF to a final protein concentration 10 mg mL^−1^ for 10 min on ice and then diluted 1:10 with the buffer containing 1% Triton X100, 160 mM NaCl, 20 mM Tris-HCl, pH 7.5, 10% glycerol, and 2 mM PMSF, and incubated at 4 °C for 15 min. The unsolubilized material was removed by centrifugation and the supernatant was incubated with anti-FLAG M2 Affinity Gel for 1.5 h at 4 °C. The beads were washed with wash buffer containing 0.1% Triton X100, 160 mM NaCl, 20 mM Tris-HCl, pH 7.5, 10% glycerol, and 2 mM PMSF. Bound proteins were eluted with 0.1 M glycine pH 2.8.

### Radiolabeling of proteins and import into mitochondria

For the synthesis of radiolabeled Atp10 and Ina22 fragments, corresponding open reading frames or their parts were amplified using a forward primer containing the SP6 polymerase binding site (Supplementary Table 2). Constructs were in vitro transcribed using mMESSAGE mMACHINE SP6 Kit (Life Technologies) and the obtained RNAs were in vitro translated with Flexi Rabbit Reticulocyte Lysate System (Promega) in the presence of [^35^S] methionine. Import into isolated mitochondria was performed according to a published procedure^37^. In brief, reaction was performed in import buffer (250 mM sucrose, 10 mM MOPS/KOH pH 7.2, 80 mM KCl, 2 mM KH_2_PO_4_, 5 mM MgCl_2_, 5 mM methionine, 3% fatty acid-free BSA, 2 mM ATP, and 2 mM NADH). The reaction was stopped with 1 µM valinomyin, 8 µM antimycin A, and 20 µM oligomycin and samples were treated with 20 µg mL^−1^ Proteinase K (PK) for 10 min on ice to digest unimported precursor proteins when needed. PK was inhibited with 2 mM phenylmethylsulphonyl fluoride (PMSF) for 10 min on ice; mitochondria were pelleted, washed with 250 mM sucrose, 20 mM MOPS pH 7.2, 1 mM EDTA and further analyzed by SDS-PAGE and autoradiography or used for co-isolation experiments.

### Chemical crosslinking

For protein crosslinking, mitochondria were solubilized in crosslinking buffer (20 mM HEPES pH 7.4, 100 mM NaCl) to a final protein concentration 2 mg mL^−1^. The cross-linker reagents diluted in DMSO were added to a final concentration 250 μM and samples were incubated on ice for 30 min. Afterwards, the cross-linker was quenched for 15 min with 100 mM glycine pH 8.0 and mitochondria were reisolated for further analyses.

### Blue-Native PAGE

Protein complexes were analyzed by BN-PAGE as published^38,39^. In brief, mitochondria were solubilized in buffer containing either 0.6% DDM or 1% digitonin, 20 mM Tris-HCl pH 7.4, 150 mM NaCl, 10% Glycerol, 1 mM PMSF on ice for 15 min. Unsolubilized material was removed by centrifugation at 16,000×*g* at 4 **°**C for 10 min and the supernatant was mixed with BN-PAGE loading buffer (0.5% Coomassie Brilliant Blue G-250, 50 mM 6-aminocaproic acid, 10 mM Bis-Tris-HCl pH 7), and loaded onto a gradient gel of desired percentage with 4% stacking gel. Electrophoresis was performed using the SE600 Ruby System (GE Healthcare) at 4 **°**C, starting with the voltage of 200 V, 15 mA for 1 h, and then shifted to 600 V.

### Sample preparation for LC-MS analysis

Following BN-PAGE separation of Atp10^ProtA^-containing protein complexes purified from Atp10^ProtA^, Atp10^ProtA^ *ina22Δ*, and wild-type mitochondria as well as Ina22^FLAG^-containing protein complexes purified from Ina22^FLAG^ and wild-type mitochondria, proteins were visualized using colloidal Coomassie Brilliant Blue and individual gel lanes were cut into 14 slices. Gel slices were washed and destained by alternating incubation steps in solution A (10 mM NH_4_HCO_3_) and B (10 mM NH_4_HCO_3_/50% acetonitrile [ACN]) for 10 min at RT. Thiol moieties were reduced by incubation in solution A containing 10 mM dithiothreitol for 30 min at 56 °C followed by alkylation with 50 mM iodoacetamide for 30 min at RT in the dark. Protein digestion was performed in solution A containing 60 ng of trypsin per slice at 37 °C overnight. Peptides were eluted with 0.05% trifluoroacetic acid (TFA)/50% (v/v) ACN and subsequently dried in vacuo. For MS analysis, peptides were resuspended 15 µL 0.1% TFA. Atp23-containing protein complexes immunopurified from wild type and *ina22Δ* mitochondria in biological triplicates were subjected to in-solution digestion as described previously^40^.

### LC-MS and data analysis

Peptides derived from in-gel digestion were analyzed by LC-MS using an UltiMate 3000 RSLCnano HPLC system (Thermo Fisher Scientific, Dreieich, Germany) directly coupled to an Orbitrap Elite mass spectrometer (Thermo Fisher Scientific, Bremen, Germany). Peptides were washed and preconcentrated on a PepMap^TM^ C18 precolumn (5 mm × 300 µm inner diameter; Thermo Scientific). For peptide separation, an AcclaimPepMap^TM^ RSLC column (50 cm × 75 µm inner diameter; pore size, 100 Å; particle size, 2 µm) was used at a flow rate of 250 nL min^−1^ and at 43 °C with a binary mobile phase consisting of 0.1% formic acid/4% DMSO (solvent A) and 0.1% formic acid/4% DMSO/48% methanol/30% ACN (solvent B). Peptides were eluted using an 80 min gradient (solvent B: 1% from 0–20 min, 1–65% from 20 to 54 min, 65–95% from 54 to 59 min, 95% for 5 min followed by a 16 min equilibration phase). The Orbitrap Elite was equipped with a Nanospray Flex ion source with DirectJunction and a fused silica emitter (Thermo Scientific). The spray voltage was 1.5 kV and the capillary temperature 200 °C. The mass spectrometer was externally calibrated using standard compounds. The following parameters were used for MS analysis: MS survey scans (*m*/*z* 370–1700) were acquired in the orbitrap at a resolution of 120,000 (at *m*/*z* 400). Automatic gain control was set to 1 × 10^6^ ions and the maximum injection time to 200 ms. Top *n* spacing was set to 15 for low-energy collision-induced dissociation of multiply charged precursor peptides in the linear ion trap applying a normalized collision energy of 35%, an activation *q* of 0.25, an activation time of 10 ms, an AGC of 5 × 10^3^, and a max. ion time of 150 ms. The dynamic exclusion time for previously fragmented precursors was set to 45 s. Peptides derived from in-solution digestion were analyzed using an UltiMate 3000 RSLCnano HPLC system coupled to an Q Exactive instrument (Thermo Fisher Scientific, Bremen, Germany) as described previously^40^. Peptides were eluted using a 180 min gradient (0.1% formic acid/86% acetonitrile: 4% from 0 to 5 min, 4–39% from 5 to 140 min, 39–54% from 140 to 155 min, 54–95% from 155 to 160 min, 95% for 5 min followed by a 15 min equilibration phase).

For protein identification and label-free quantification, raw files were processed using MaxQuant^41^ (version 1.5.3.12) and Andromeda^42^. The Saccharomyces Genome Database (SGD; www.yeastgenome.org) was used for the database search. Database searches were performed with mass tolerances of 4.5 ppm for precursors and 0.5 Da for fragment ions. “trypsin/P” was set as enzyme specificity and ‘Max. missed cleavages’ was set to 3. Acetylation of protein N termini and oxidation of methionine were considered as variable and carbamidomethylation of cysteine residues as fixed modification. The options “requantify”, “match between runs” (match from and to), “iBAQ”, and “LFQ” were enabled. “LFQ min. ratio count” was set to 1, “LFQ min. number of neighbors” and “LFQ average number of neighbors” were set to 3 and 6, respectively. Proteins were identified based on at least one unique peptide with a length of seven amino acids and a maximum mass of 4600 Da. For protein quantification, only unique peptides were considered and “Min. ratio count” was set to 1. False discovery rates on protein level and for peptide spectrum matching were set to 0.01. For analysis of output tables, Perseus^43^ (version 1.5.6.0) was used. LFQ ratios from Atp23 pulldown experiments were determined as follows: three out of three valid LFQ intensity values were required for at least one of the two compared experiments. Missing values were then imputed using standard settings. *P* values were calculated using a one-sided student’s *t* test (*n* = 3). Results of LC-MS analyses are provided in Supplementary Tables 1–3

### Miscellaneous

In-gel activity of complex V was determined as previously described^39^. In brief, a gel strip was cut from the BN-PAGE gel and incubated for 30 min in complex V calibration buffer (35 mM Tris, 220 mM glycine pH 8.3). After this, the gel was transferred to complex V staining buffer (35 mM Tris, 220 mM glycine pH 8.3, 14 mM MgSO_4_, 0.2% (w/v) Pb(NO_3_)_2_, 8 mM ATP) and incubated at 30 °C until bands appeared. TCA protein precipitation was performed according to standard procedure. SDS-PAGE, urea-PAGE, and western blotting were performed according to standard protocols. Signals were detected using HRP-coupled secondary antibodies (used in 1:10,000 dilution, Jackson ImmunoResearch, code number 111-035-144) and enhanced chemiluminescence system (GE Healthcare). No other image processing, other than cropping, scaling, and contrast adjustment using Adobe Photoshop CS6 and Adobe Illustrator CS6 was applied.

### Statistical analysis

All quantitative data are presented as mean ± standard error of the mean (SEM). “*n*” represents the number of biological replicates. Quantifications were performed using ImageQuant TL (GE Healthcare) using a rolling ball background substraction. Number of replicates, controls, and statistical tests are in accordance with published studies employing comparable techniques and are generally accepted standards in the field. Statistical tests to determine sample size were not employed.

### Data availability

The MS proteomics data have been deposited to the ProteomeXchange Consortium via the PRIDE^44^ partner repository with the data set identifiers PXD006249 (Atp10^ProtA^ affinity chromatography experiments), PXD007146 (Atp23-immunopurification experiments) and PXD007155 (Ina22^FLAG^ immunopurification experiments). The remaining data are available within the article and its Supplementary Information files and from the corresponding author on reasonable request.

## Electronic supplementary material

Supplementary Information

Description of Additional Supplementary Files

Supplementary Data 1

Supplementary Data 2

Supplementary Data 3
