# Supplementary material for: INA complex liaises the F1Fo-ATP synthase membrane motor modules
Source: Nat Commun. 2017 Nov 1;8:1237. doi: 10.1038/s41467-017-01437-z (PMC5665977; doi:10.1038/s41467-017-01437-z)
Supplement: Supplementary file 1 — Supplementary Information [file 41467_2017_1437_MOESM1_ESM.pdf]

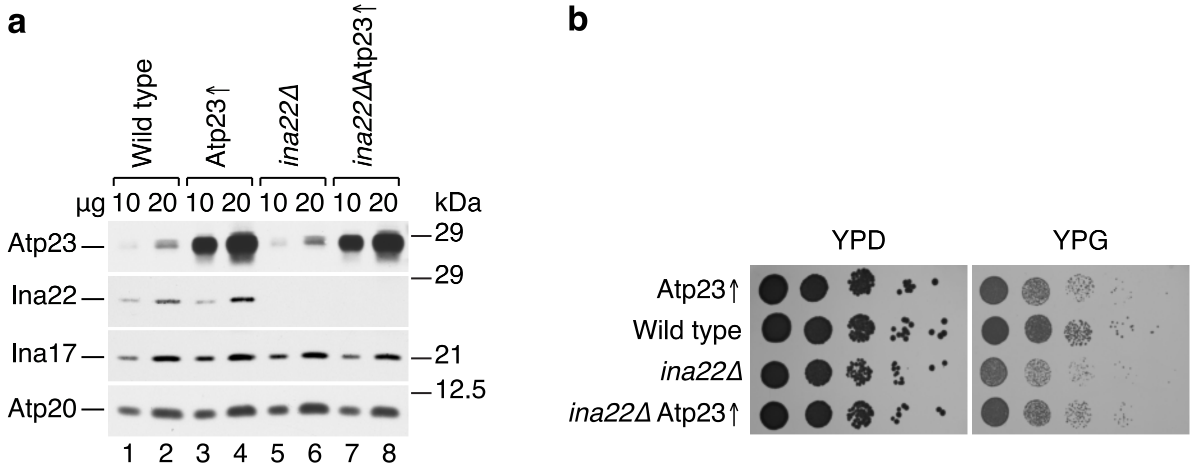

Supplementary Figure 1. Related to Figure 2

a Steady-state protein levels in wild type and mutant mitochondria were analyzed by SDS-PAGE and Western blotting with indicated antibodies.
b Serial dilution of wild type and mutant yeast cells was spotted on YPD or YPG plates. The plates were grown at 37°C for 2-5 days.

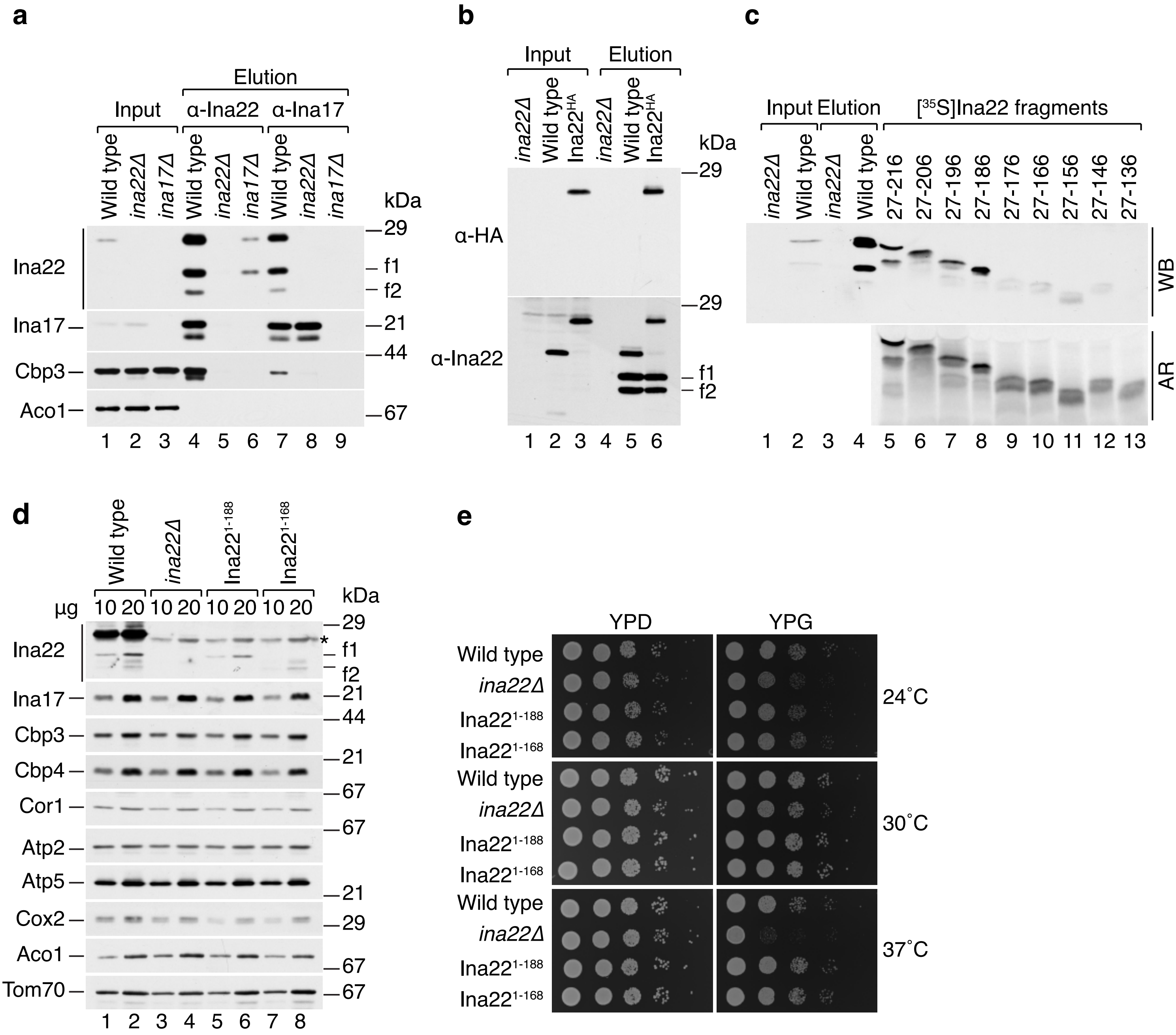

Supplementary Figure 2. Related to Figure 3

a Wild type, *ina22Δ* and *ina17Δ* mitochondria were solubilized and protein complexes were co-immunoprecipitated with anti-Ina22 and anti-Ina17 antibodies. Input and elution fractions were analyzed by SDS-PAGE and Western blotting with indicated antibodies. Input = 1% of elution. f1, f2 – Ina22 fragments.

b *ina22Δ*, wild type and $Ina22^{HA}$ mitochondria were subjected to co-immunoprecipitation with anti-Ina22 antibodies. Input and elution fractions were analyzed by SDS-PAGE and Western blotting with either anti-HA or anti-Ina22 antibodies. Input = 1% of elution. f1, f2 – Ina22 fragments.

c Ina22 was immunoprecipitated from wild type and *ina22Δ* mitochondria with anti-Ina22 antibodies and the molecular weight of its fragments was assessed by comparing their electrophoretic mobility (lanes 1-4) to the mobility of in vitro generated radio-labeled Ina22 fragments (lanes 5-13). Samples were analyzed by SDS-PAGE, digital autoradiography (AR) and Western blotting (WB).

d Mitochondrial proteins in wild type and mutant mitochondria were analyzed by SDS-PAGE and Western blotting with the indicated antibodies. The asterisk (*) indicates a cross-reacting band. f1, f2 – Ina22 fragments.

e Serial dilutions of yeast cells were spotted on YPD or YPG plates. The plates were grown at the indicated temperatures for 2-5 days.

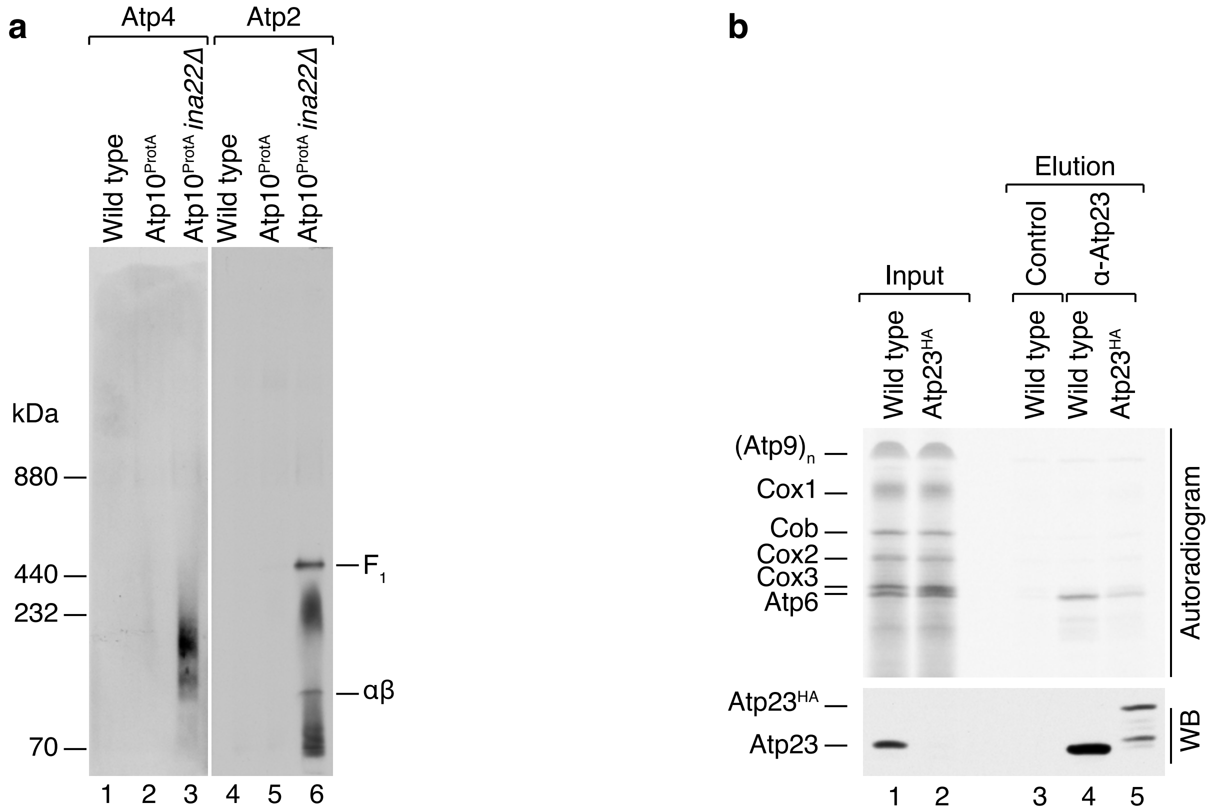

Supplementary Figure 3. Related to Figure 4

a Wild type, Atp10^{ProtA} and Atp10^{ProtA}*ina22Δ* mitochondria were solubilized and subjected to IgG affinity chromatography. Purified complexes were eluted via TEV-protease cleavage and analyzed by BN-PAGE and Western blotting with indicated antibodies. Aβ – dimer containing Atp1 and Atp2.
b Wild type and Atp23^{HA} mitochondria were subjected to in organello labeling for 20 minutes and immunoprecipitation with control or anti-Atp23 antibodies. Input and elution fractions were analyzed by Urea SDS-PAGE, Western blotting (WB) and autoradiography. Input = 2% of elution

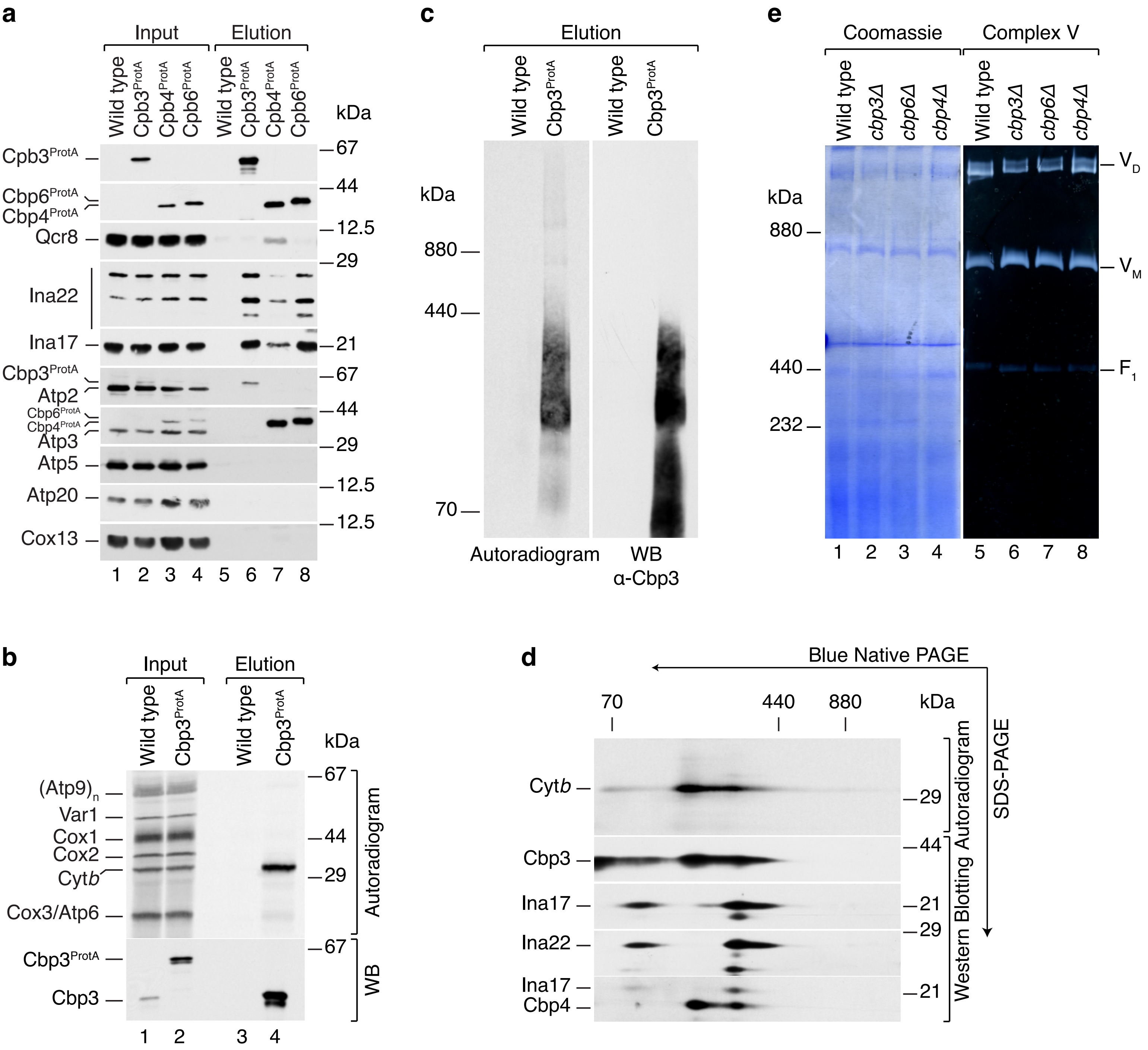

Supplementary Figure 4. Related to Figure 6

The INA complex associates with cytochrome *b* biogenesis factors

a Protein complexes were isolated via IgG affinity chromatography from Cbp3^{ProtA}, Cbp4^{ProtA}, Cbp6^{ProtA} and control wild type mitochondria and analyzed by SDS-PAGE and Western blotting with indicated antibodies. Input = 1% of elution.
b Mitochondrial-encoded proteins were radiolabeled in organello and protein complexes were isolated from Cbp3^{ProtA} and wild type mitochondria via IgG affinity chromatography and TEV-cleavage. Input and elution fractions were analyzed by SDS-PAGE, Western blotting (WB) with anti-Cbp3 antibodies, or autoradiography. Input = 1% of elution.
c Part of the elution fractions of the experiment presented in Supplementary Figure 4b was analyzed by BN-PAGE, followed by either Western blotting (WB) with anti-Cbp3 antibodies (right panel) or autoradiography (left panel).
d Part of the Cbp3^{ProtA} elution of the experiment presented in Supplementary Figure 4b was analyzed by BN-PAGE, followed by second dimension SDS-PAGE, autoradiography (upper panel) or Western blotting with the indicated antibodies.
e *cbp3Δ, cbp4Δ, cbp6Δ* and control wild type mitochondria were solubilized and analyzed by BN-PAGE followed by either gel

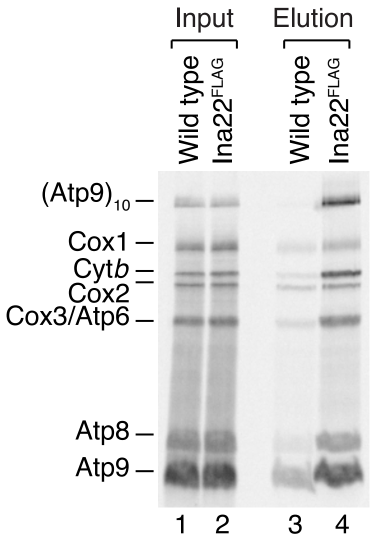

Supplementary Figure 5. Related to Figure 6

Chloramphenicol-pretreated wild type and Ina22[FLAG] mitochondria were subjected to in organello labeling for 20 min, anti-FLAG affinity chromatography and further analysis as described in Figure 6a. Input = 1% of elution.

Supplementary Figure 6.

Original scans of key Western blots and gels presented in the paper

Figure 1d

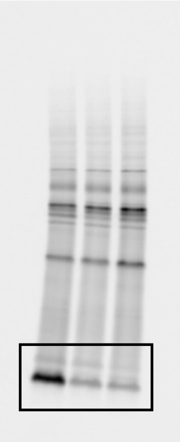

Figure 2a

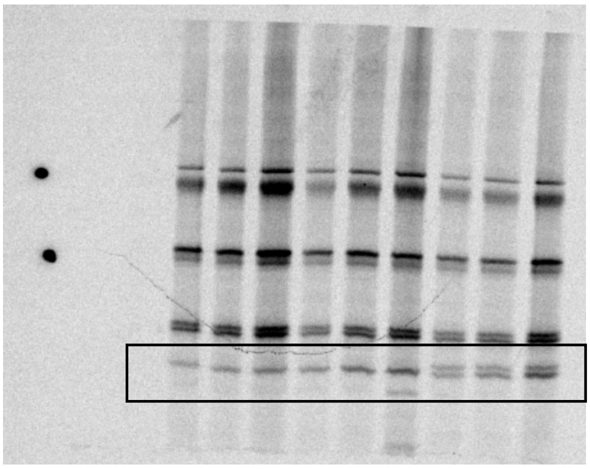

Figure 2b

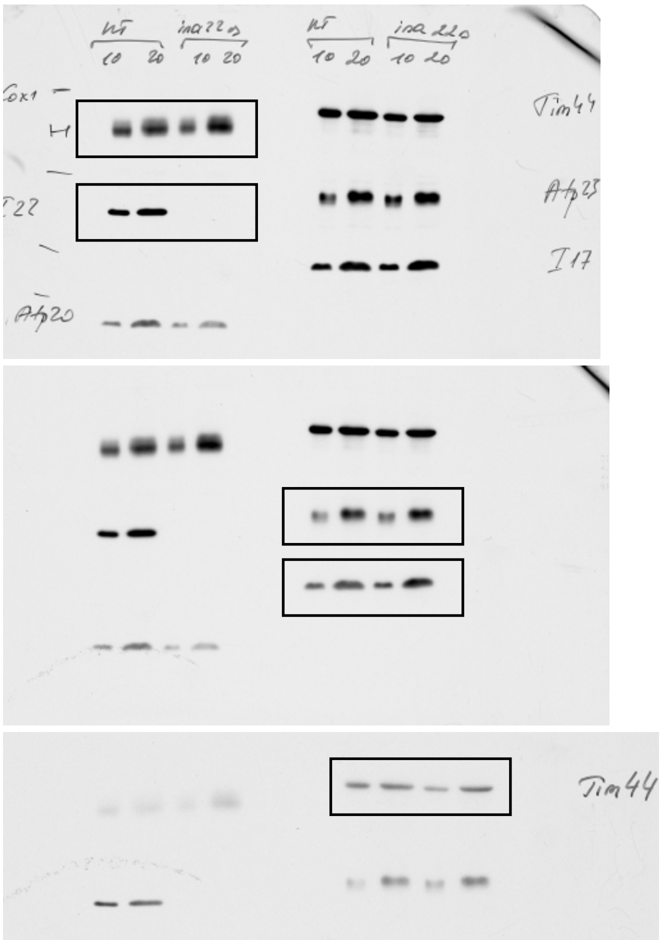

Figure 2d

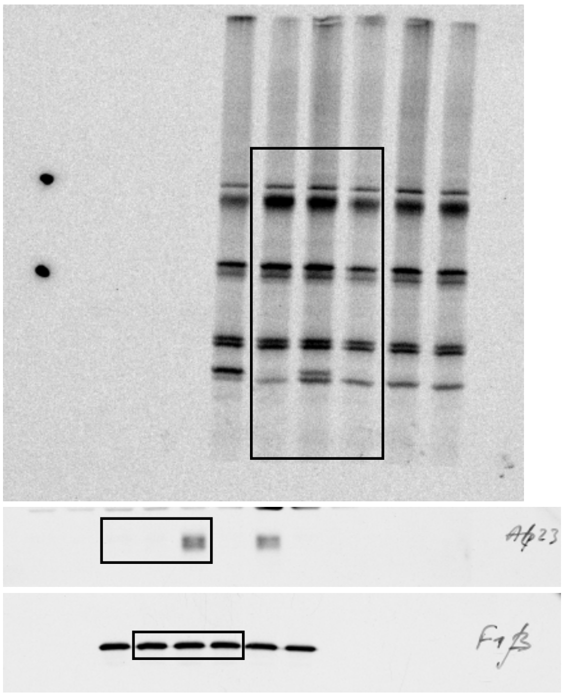

Figure 3a

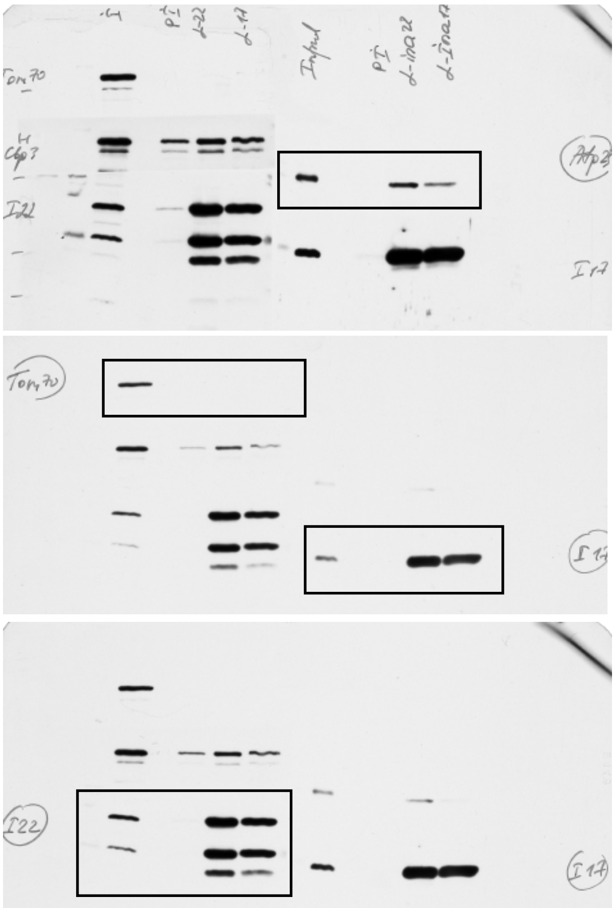

Supplementary Figure 7.

Original scans of key Western blots and gels presented in the paper

Figure 3b

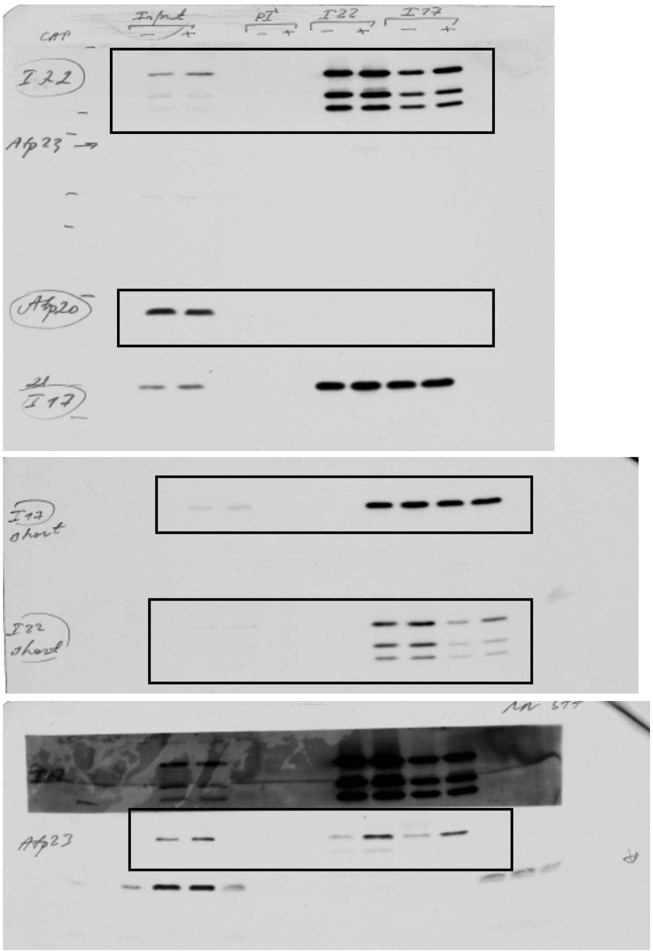

Figure 3e

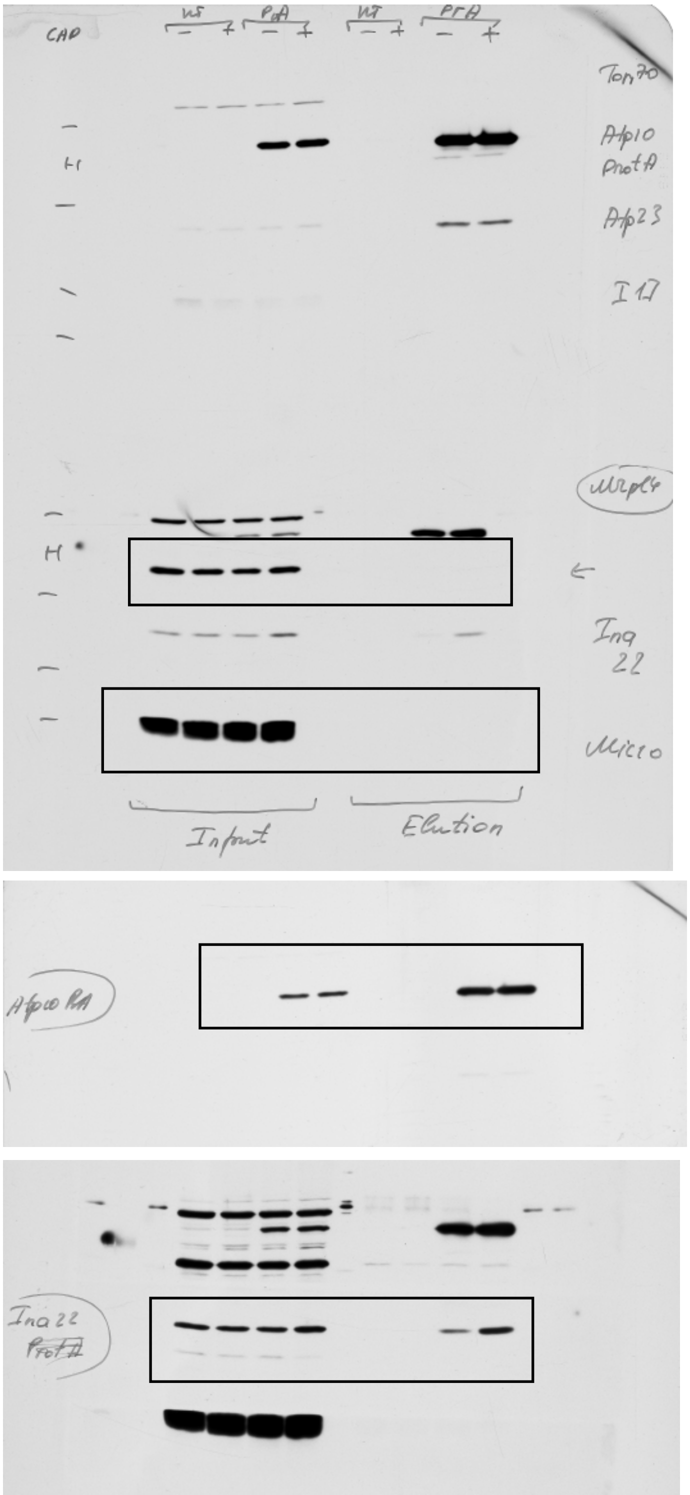

Figure 3e

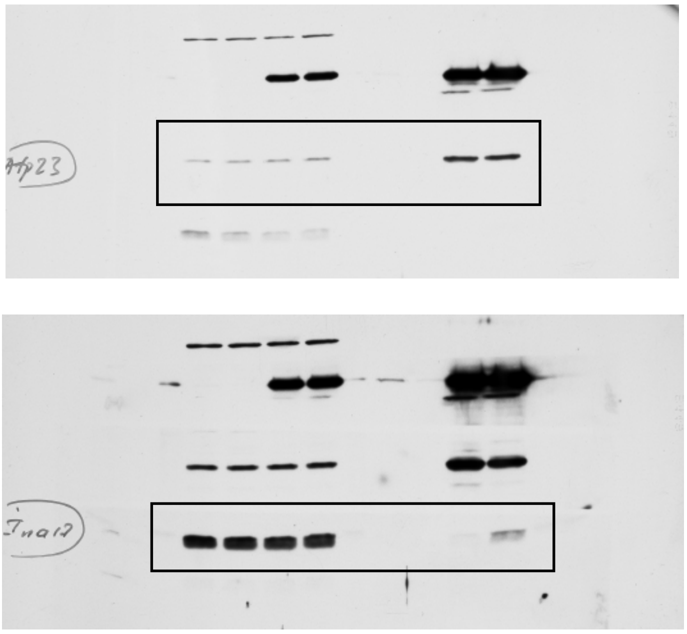

Supplementary Figure 8.

Original scans of key Western blots and gels presented in the paper

Figure 3f

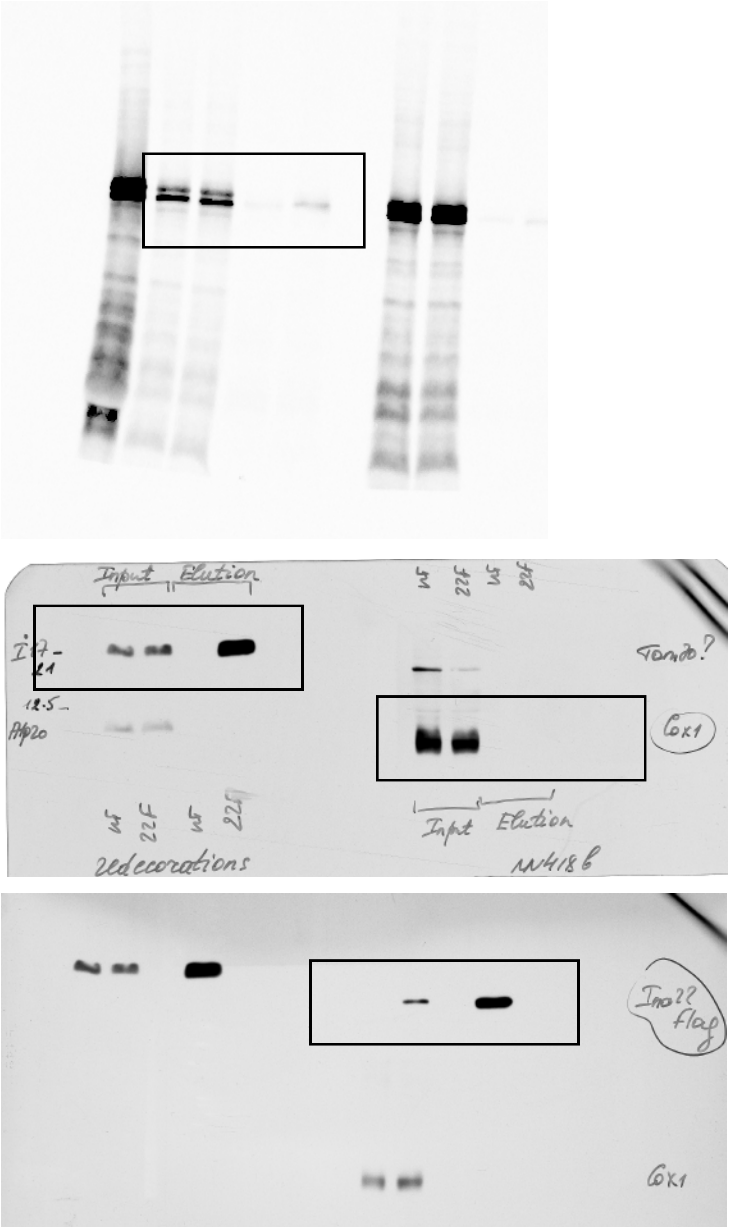

Figure 3g

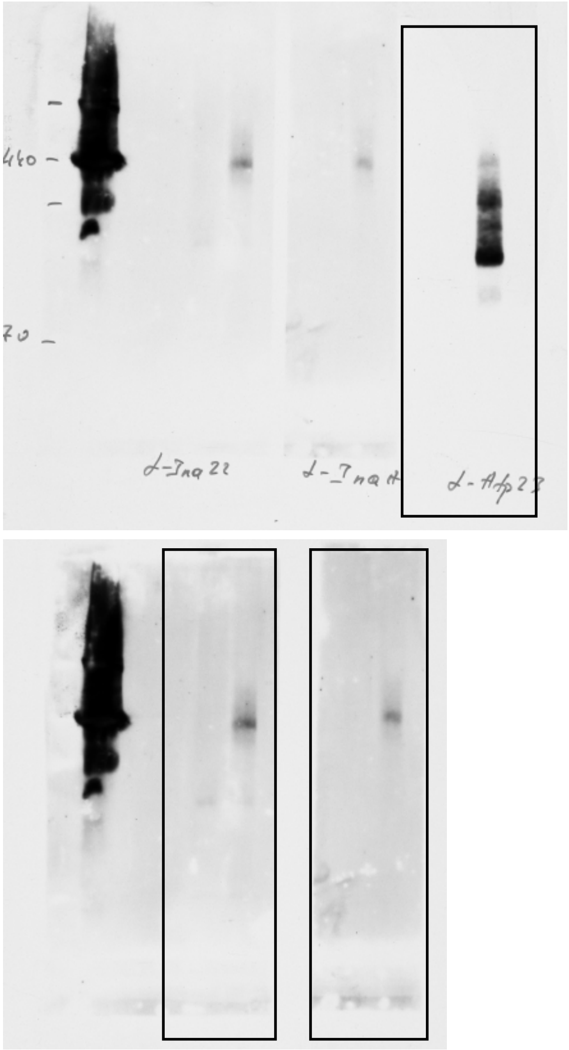

Figure 4a

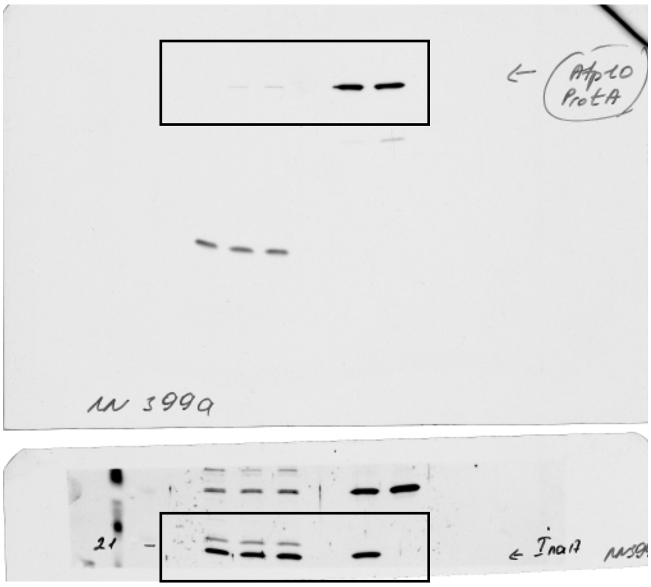

Figure 4a

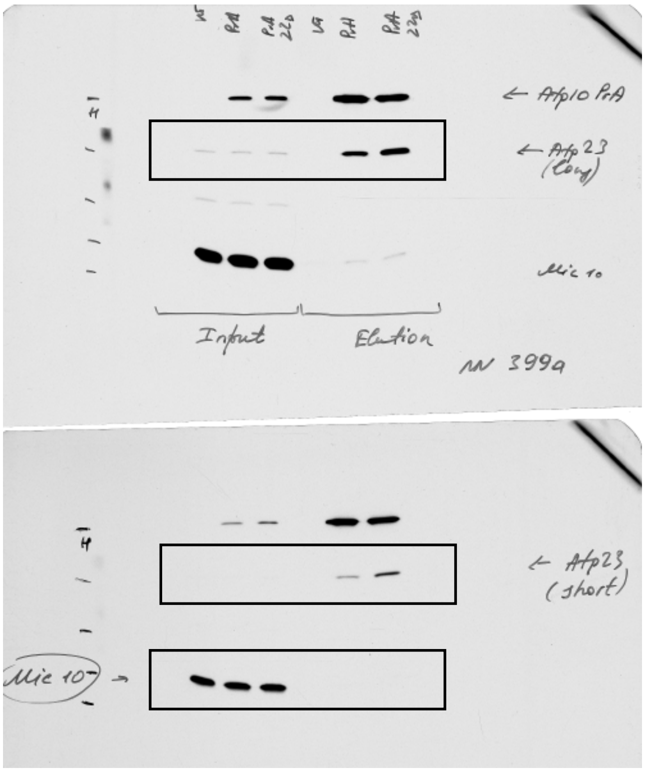

Supplementary Figure 9.

Original scans of key Western blots and gels presented in the paper

Figure 4c

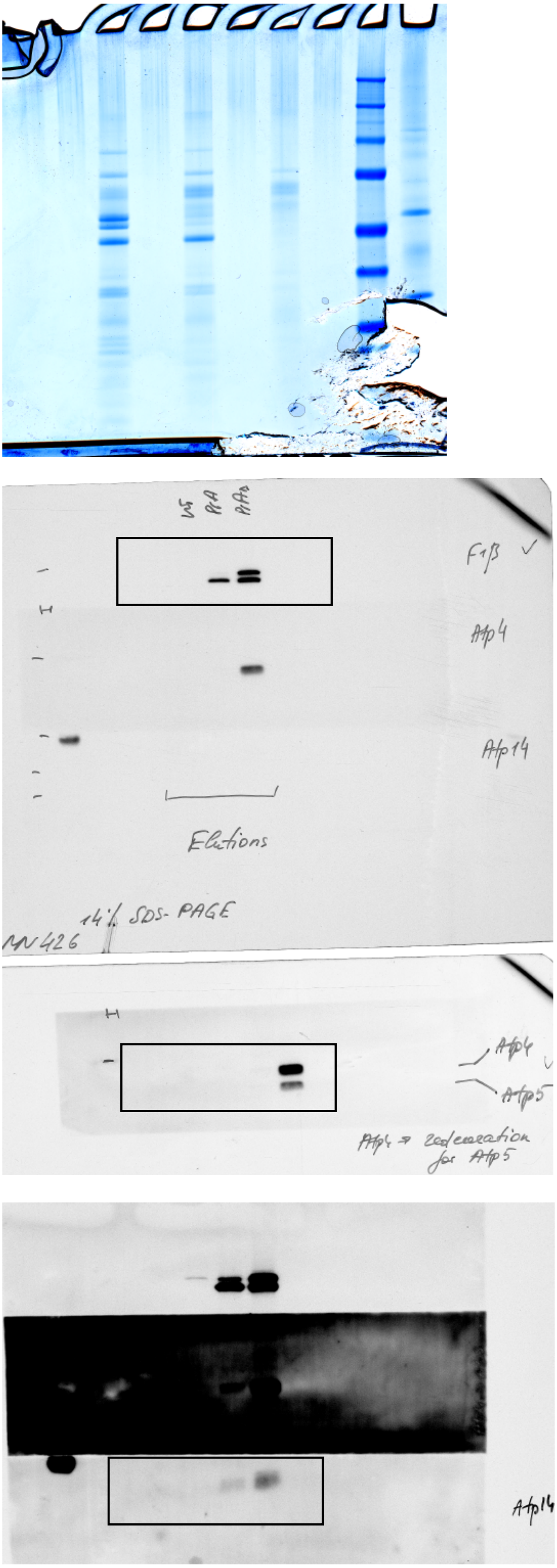

Figure 4e

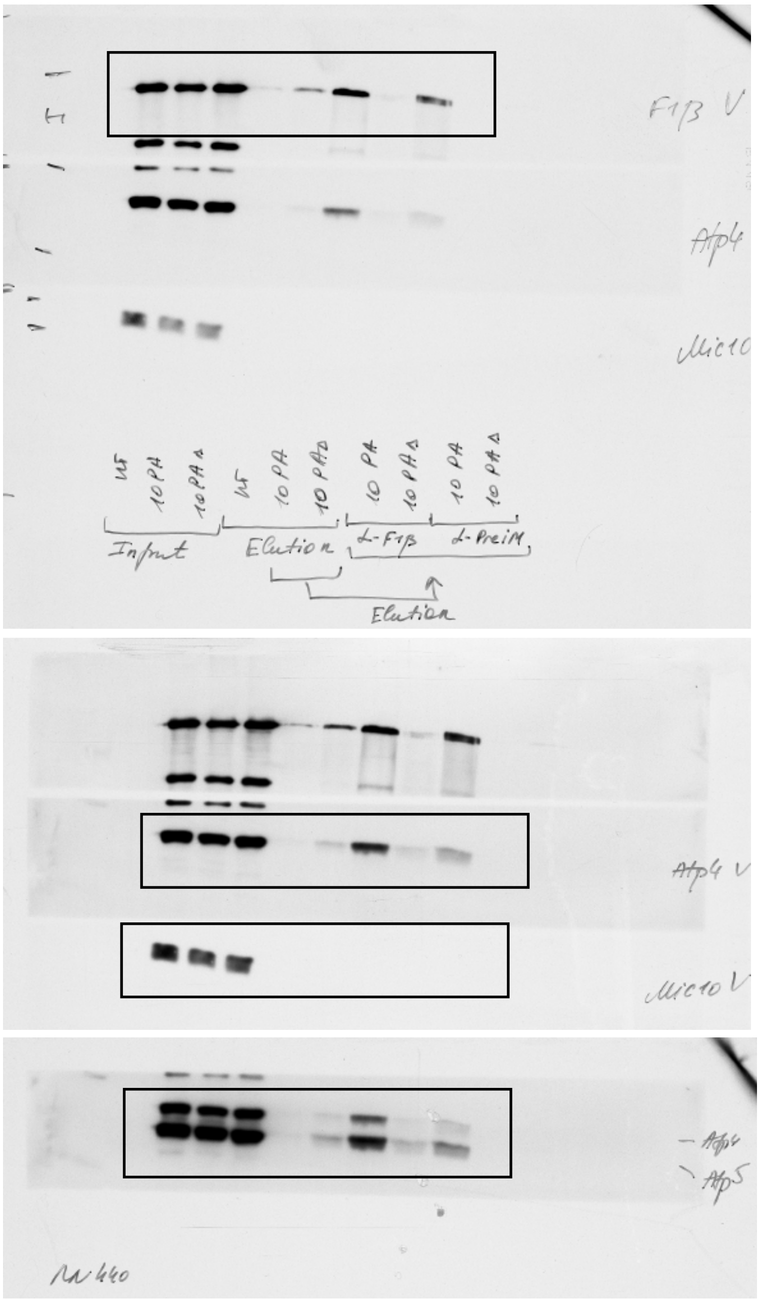

Supplementary Figure 10.

Original scans of key Western blots and gels presented in the paper

Figure 4f

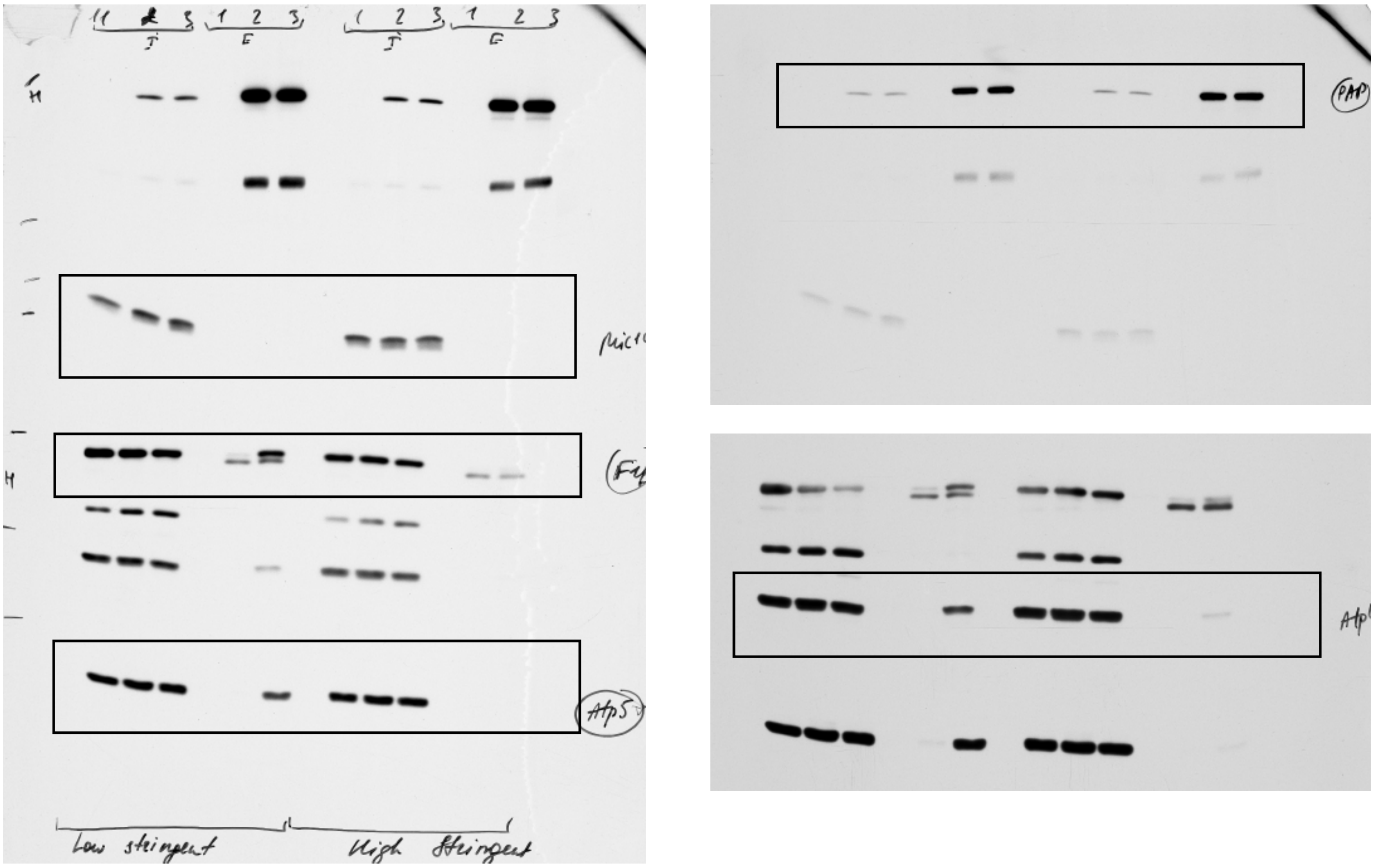

Figure 5a

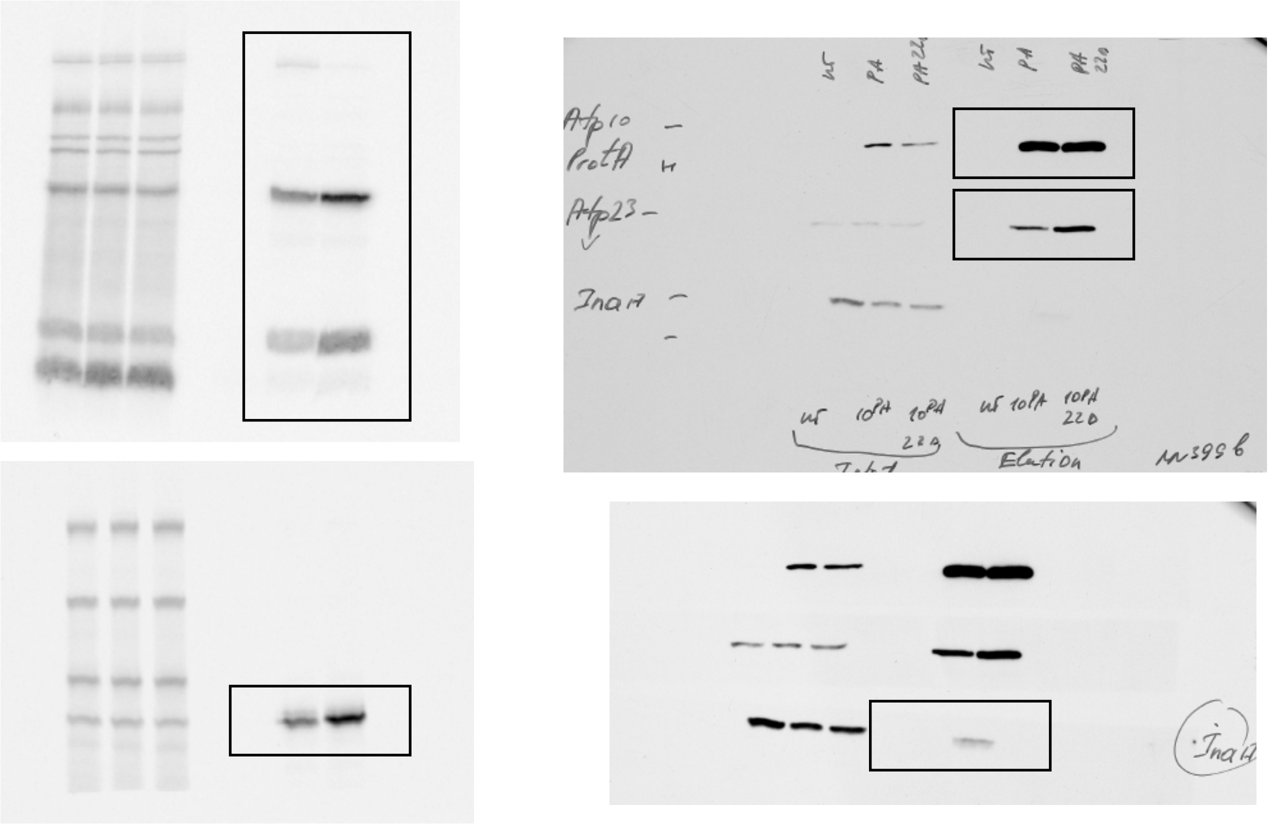

Supplementary Figure 11.

Original scans of key Western blots and gels presented in the paper

Figure 5b

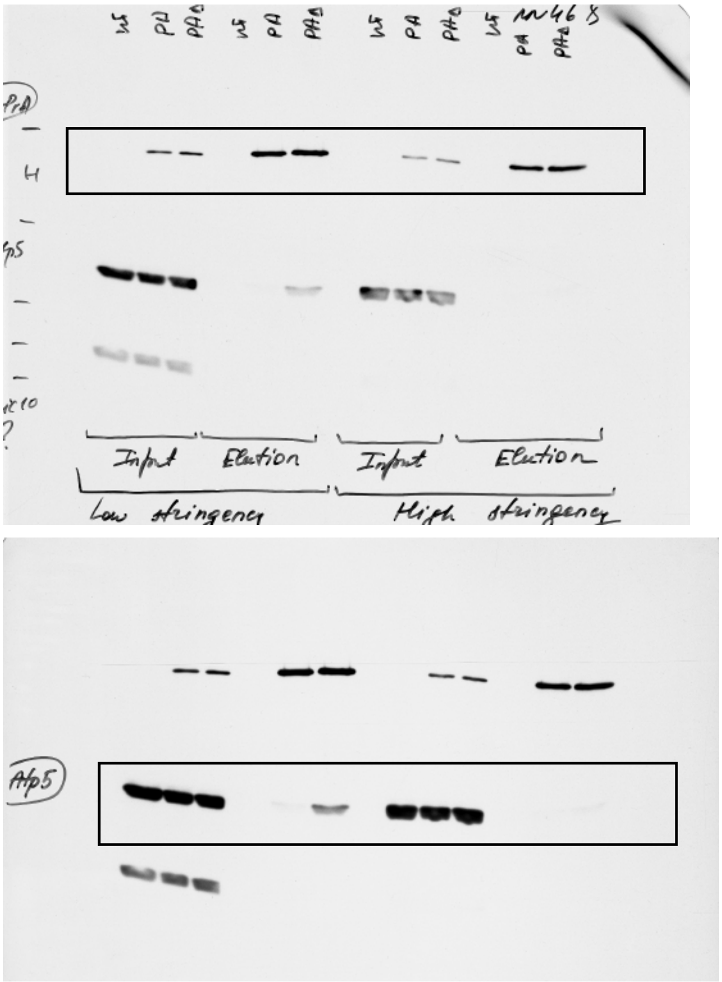

Figure 5c

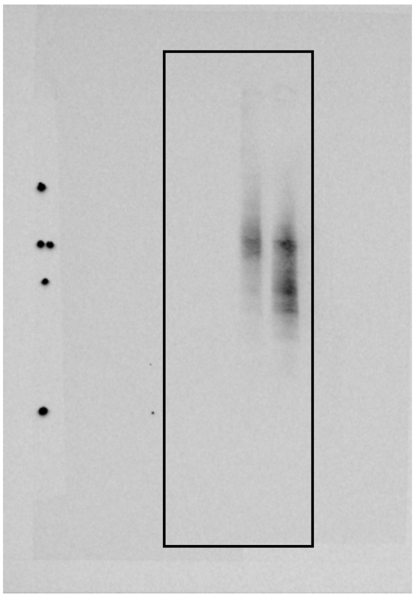

Figure 5g

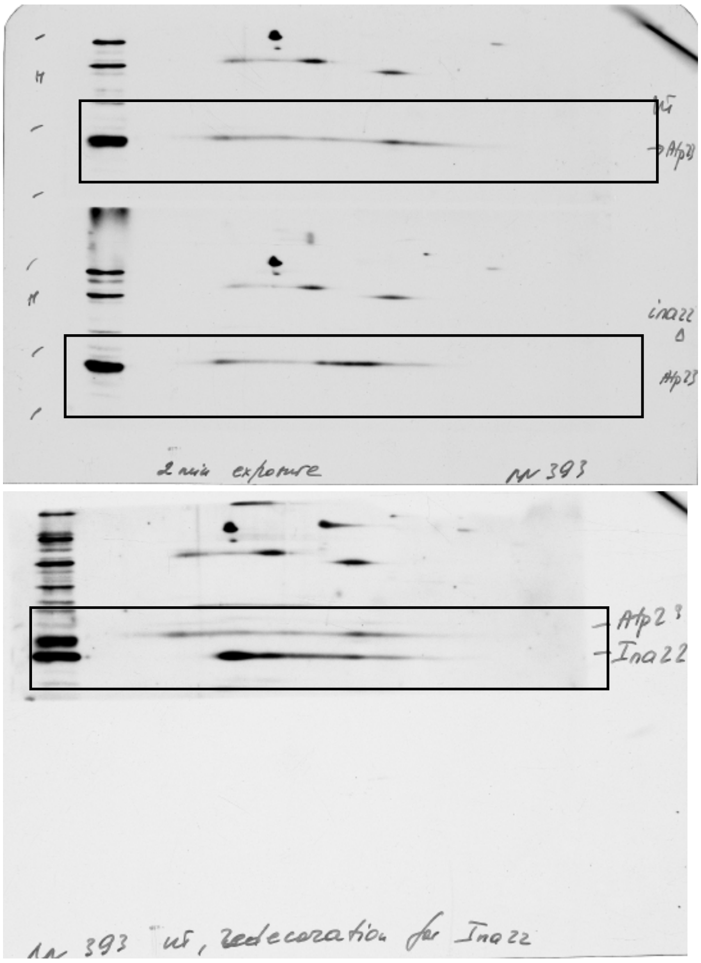

Figure 6a

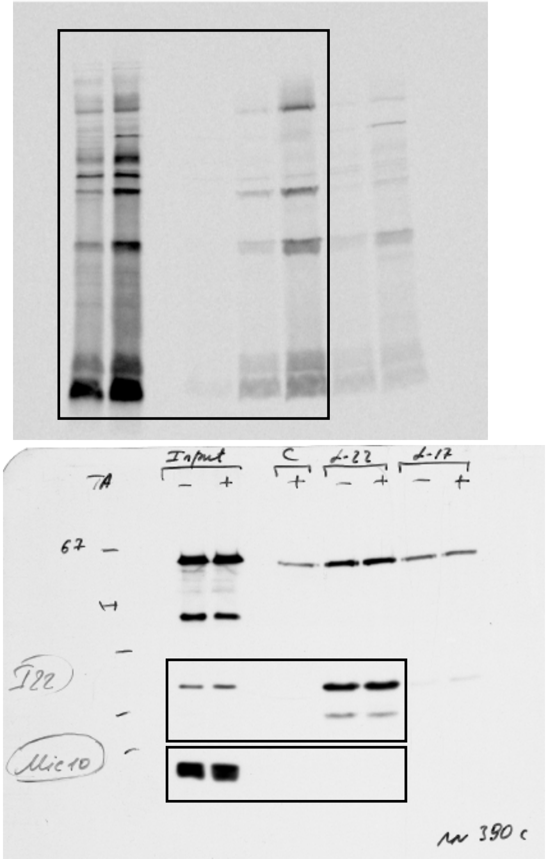

Supplementary Figure 12.

Original scans of key Western blots and gels presented in the paper

Figure 6c

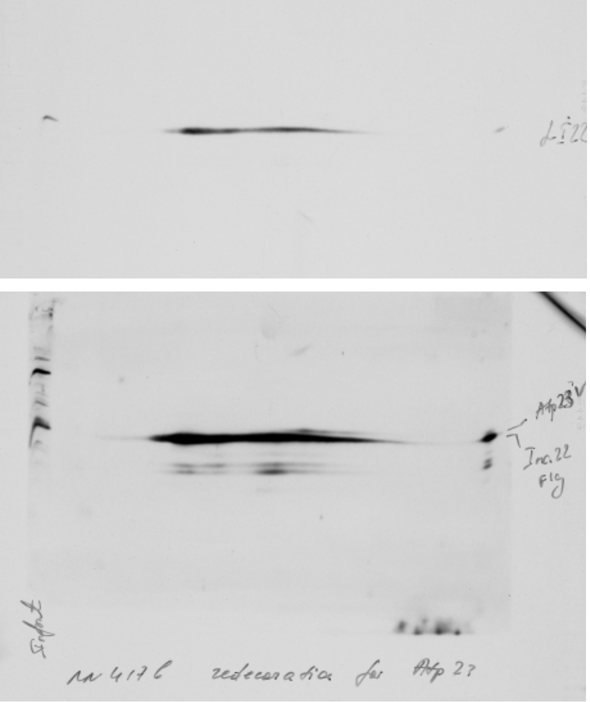

Supplementary Figure 2a

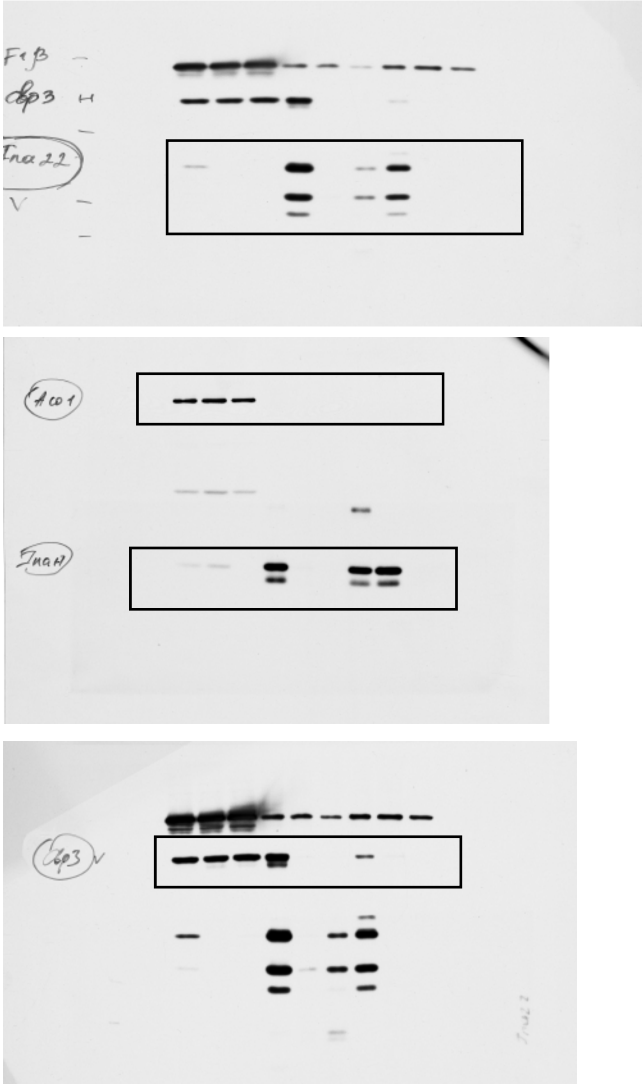

Supplementary Figure 1a

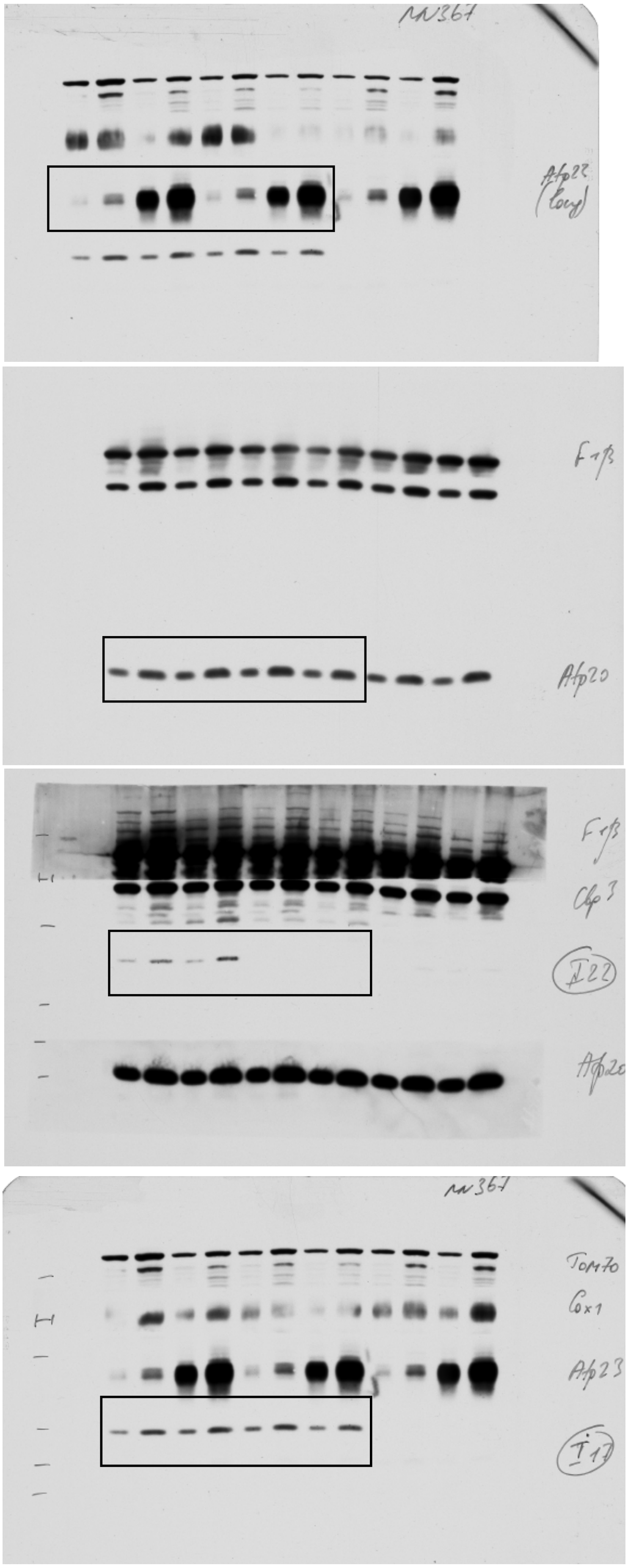

Supplementary Figure 13.

Original scans of key Western blots and gels presented in the paper

Supplementary Figure 2b

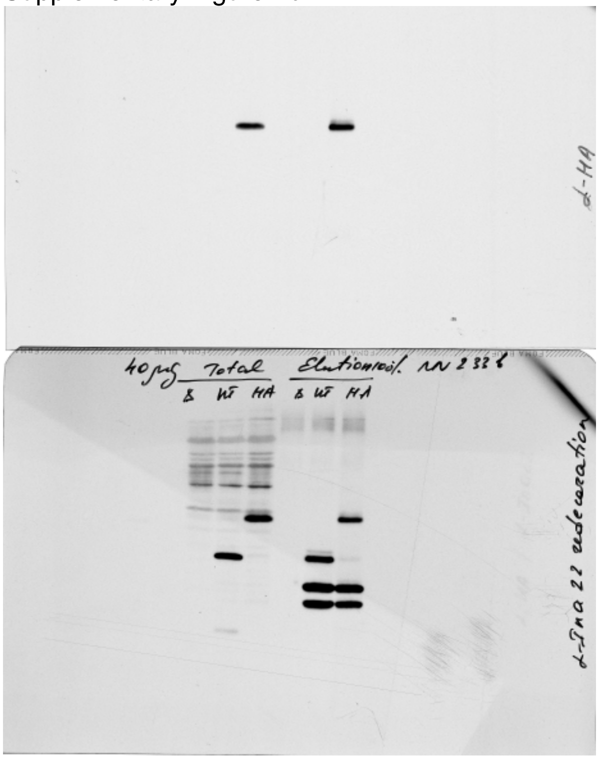

Supplementary Figure 2c

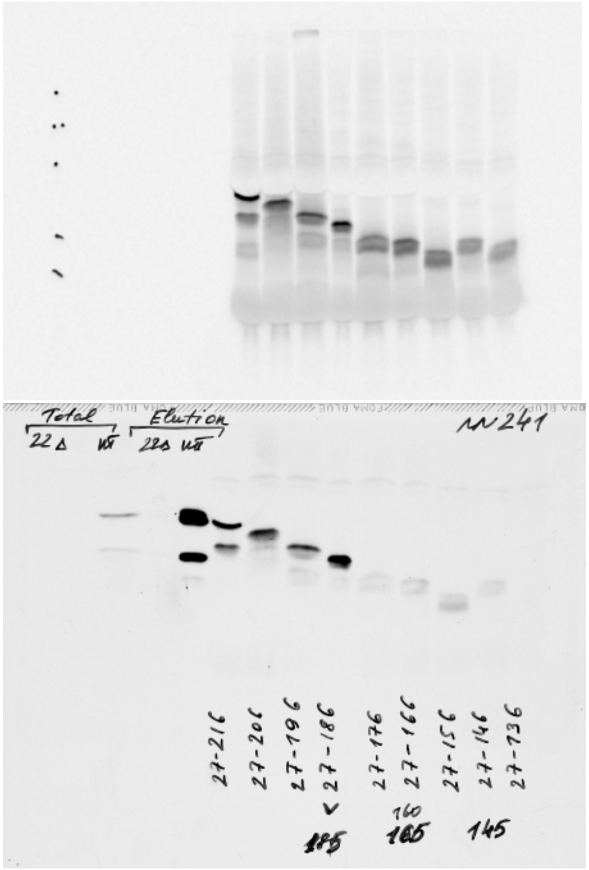

Supplementary Figure 2d

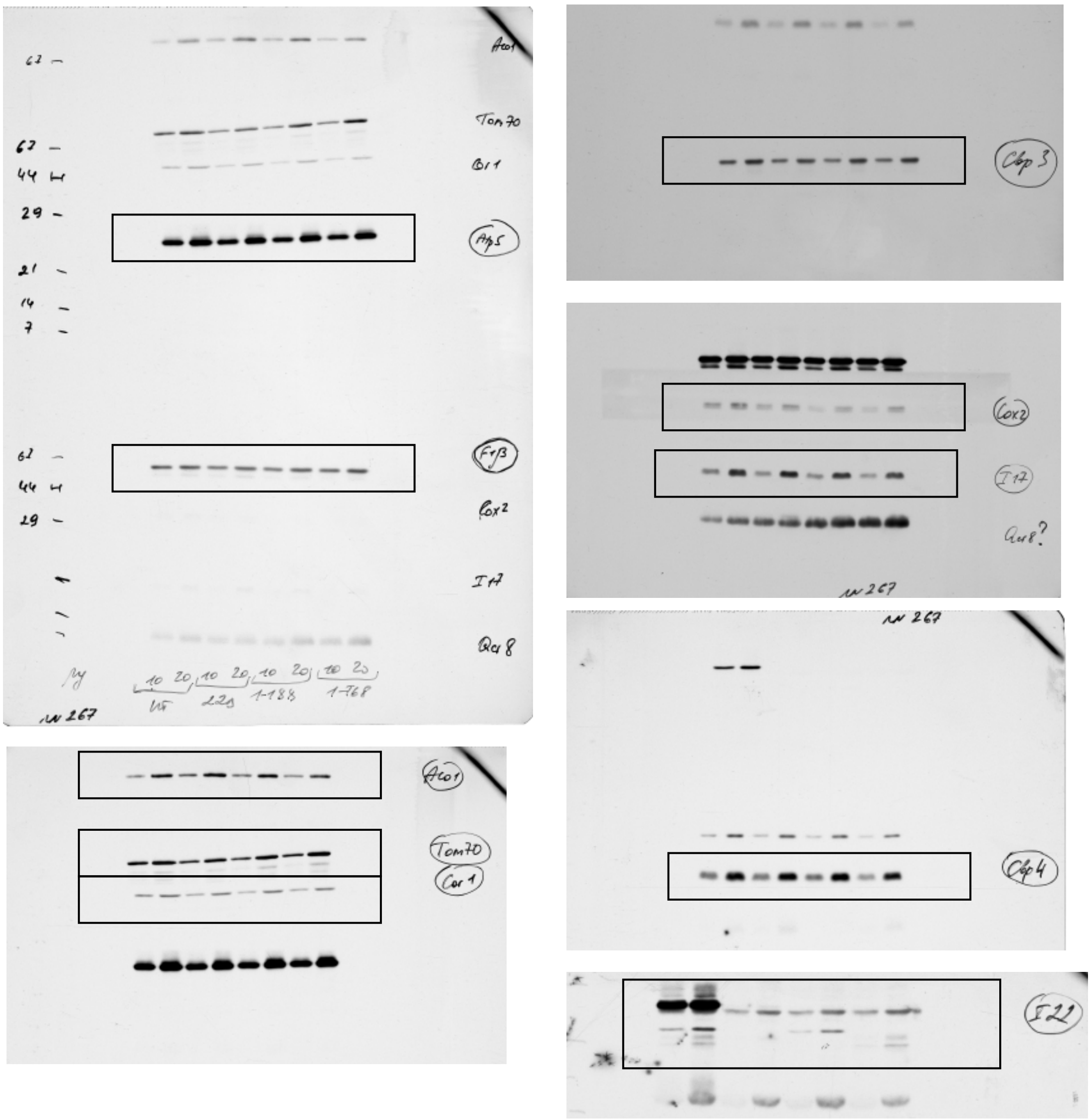

Supplementary Figure 14.

Original scans of key Western blots and gels presented in the paper

Supplementary Figure 3b

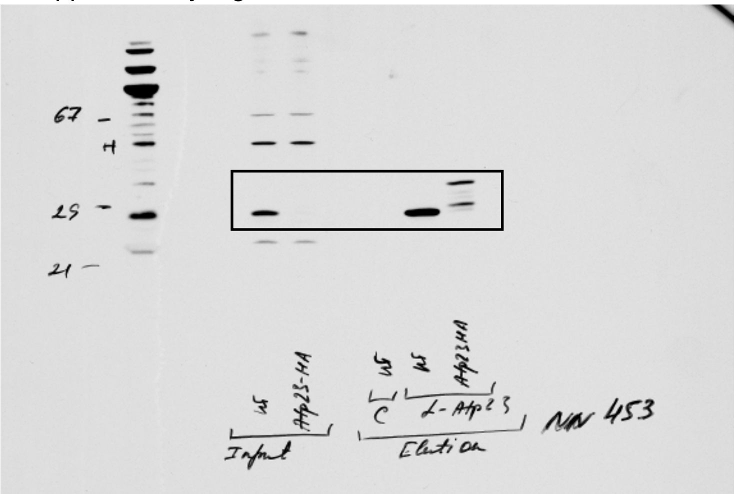

Supplementary Figure 4a

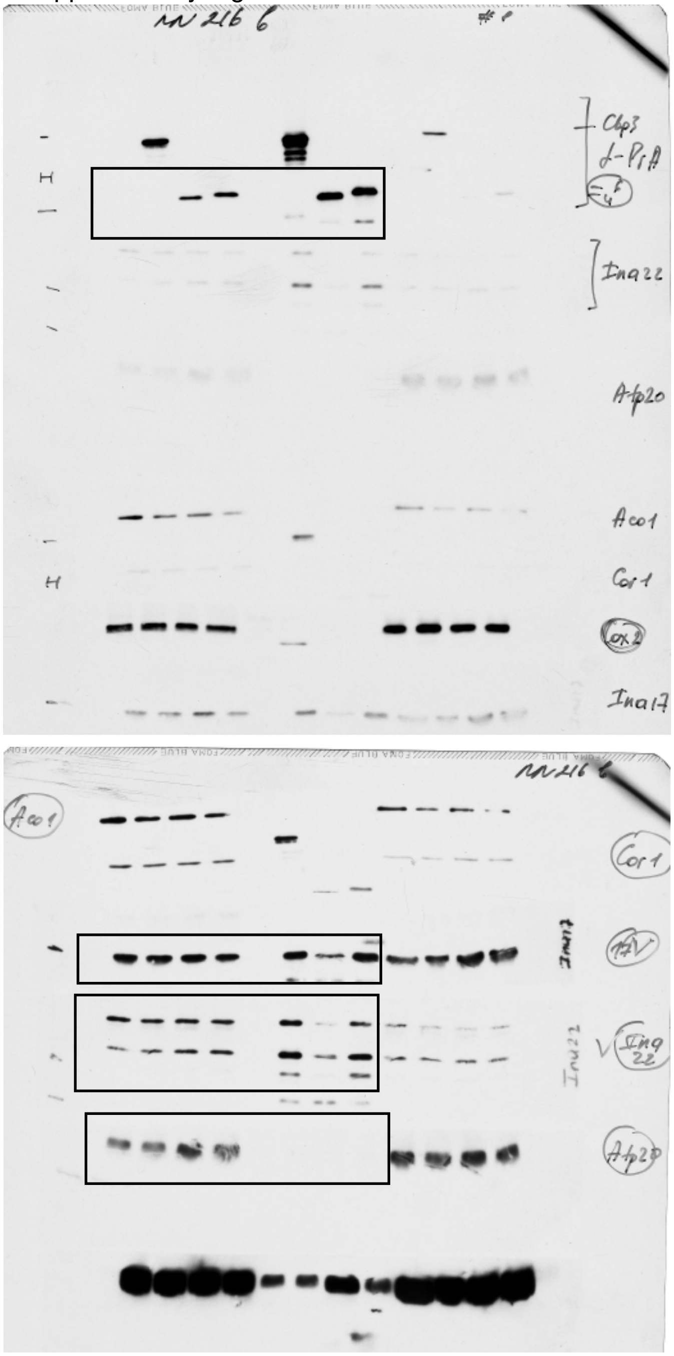

Supplementary Figure 4a

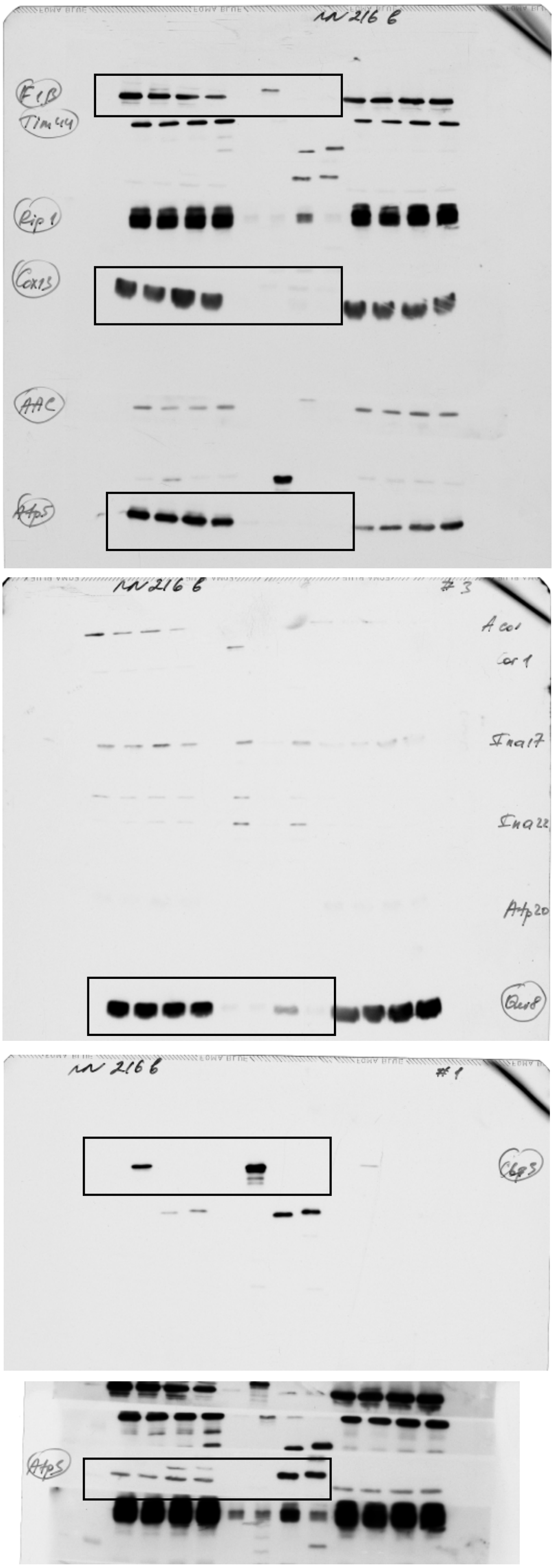

Supplementary Figure 15 .

Original scans of key Western blots and gels presented in the paper

Supplementary Figure 4b

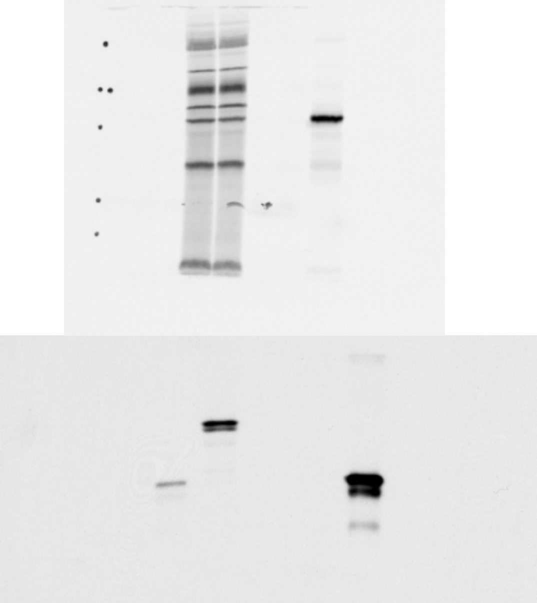

Supplementary Figure 4c

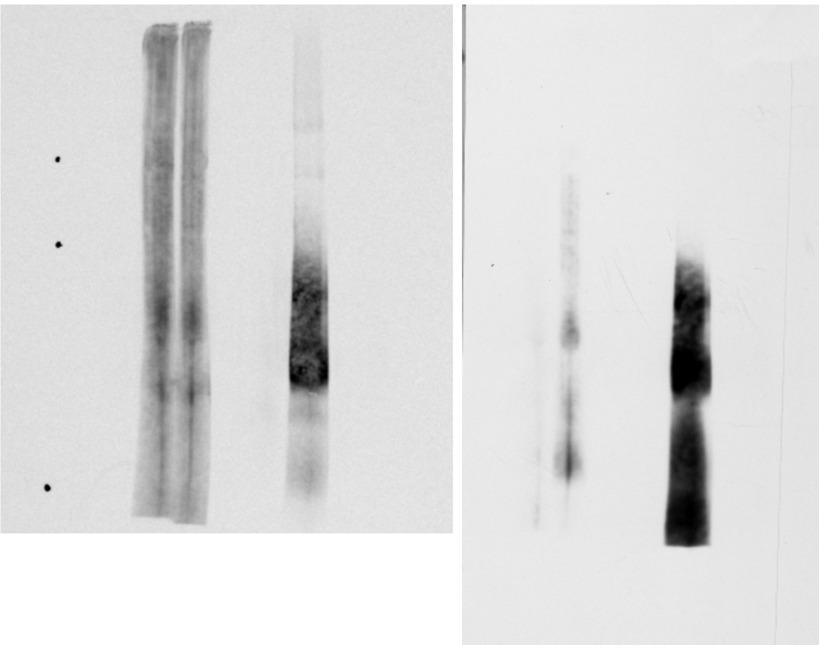

Supplementary Figure 4d

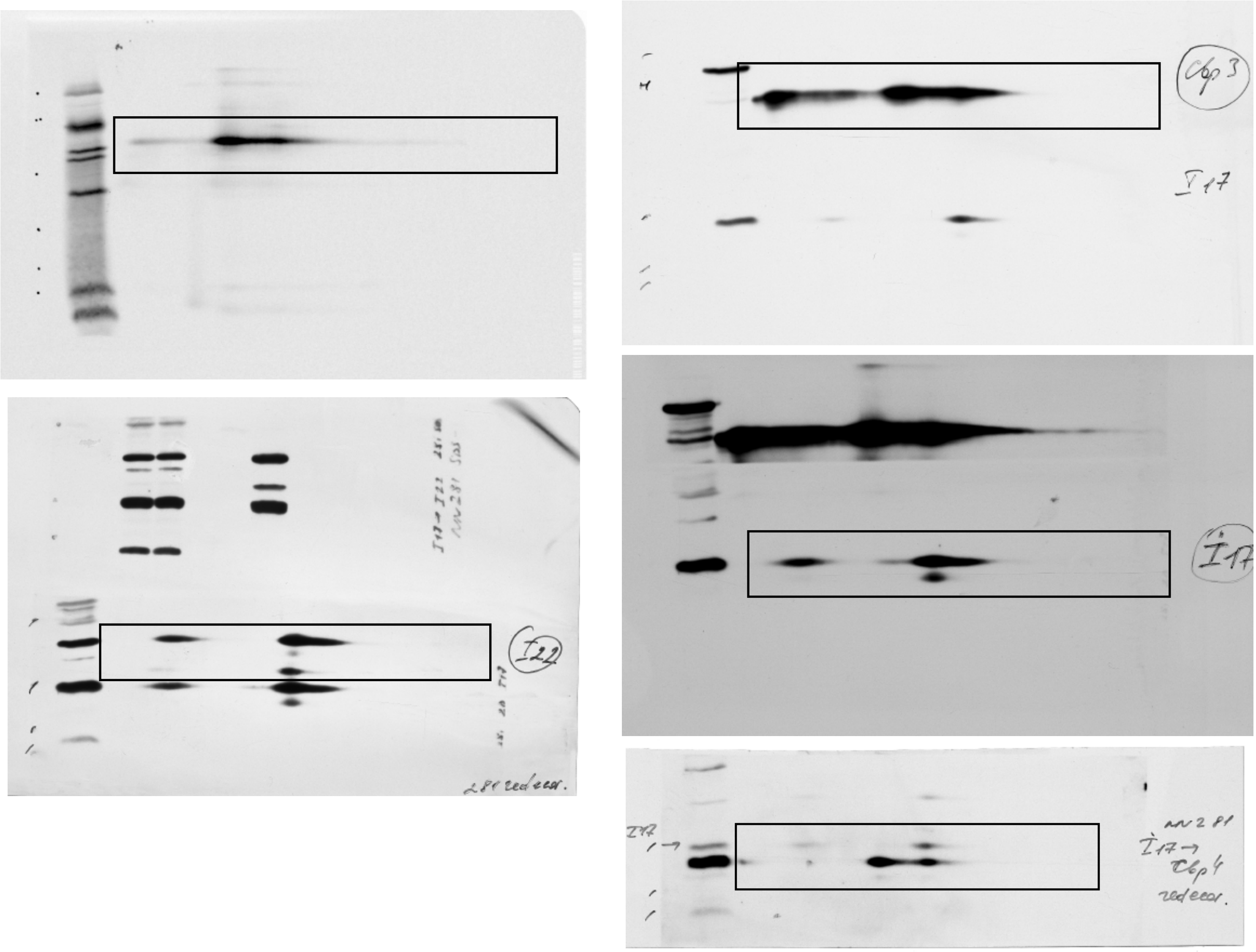

SUPPLEMENTARY INFORMATION

Supplementary Table 1

Yeast strains used is this study.

Strain	Genotype	Source or author reference
BY4741	*MATa, his3Δ1, leu2Δ, met15Δ0, ura3Δ0*	32
ina22Δ	*MATa, his3Δ1, leu2Δ, met15Δ0, ura3Δ0, INA22::kanMX4*	Euroscarf
ina17Δ	*MATa, his3Δ1, leu2Δ, met15Δ0, ura3Δ0, INA17::kanMX4*	Euroscarf
Ina22$^{1-188}$ (NNY42)	*MATa, his3Δ1, leu2Δ, met15Δ0, ura3Δ0,* $INA22^{189-216}$*::HIS3MX6*	This study
Ina22$^{1-168}$ (NNY40)	*MATa, his3Δ1, leu2Δ, met15Δ0, ura3Δ0,* $INA22^{169-216}$*::HIS3MX6*	This study
Atp23↑ (NNY106)	*MATa, his3Δ1, leu2Δ, met15Δ0, ura3Δ0, GPD:Atp23*	This study
ina22Δ Atp23↑ (NNY108)	*MATa, his3Δ1, leu2Δ, met15Δ0, ura3Δ0, INA22::kanMX4, GPD:Atp23*	This study
Ina22^{FLAG} (NNY136)	*MATa, his3Δ1, leu2Δ, met15Δ0, ura3Δ0*, ina22::INA22^{FLAG}-URA3	This study
Ina22^{HA} (OLY03)	*MATa, his3Δ1, leu2Δ, met15Δ0, ura3Δ0, ina22::*$INA22^{HA}$*-HIS3MX6*	24
Atp23^{HA} (NNY52)	*MATa, his3Δ1, leu2Δ, met15Δ0, ura3Δ0, atp23::*$ATP23^{HA}$*-HIS3MX6*	This study
Atp10^{ProtA} (NNY112)	*MATa, his3Δ1, leu2Δ, met15Δ0, ura3Δ0, atp10::ATP10-TEV-ProtA-7His-HIS3MX6*	This study
Atp10^{ProtA}*ina22Δ* (NNY113)	*MATa, his3Δ1, leu2Δ, met15Δ0, ura3Δ0, INA22::kanMX4, atp10::ATP10-TEV-ProtA-7His-HIS3MX6*	This study
Cbp3^{ProtA} (NNY23)	*MATa, his3Δ1, leu2Δ, met15Δ0, ura3Δ0, cbp3::CBP3-TEV-ProtA-7His-HIS3MX6*	This study
Cbp4^{ProtA} (NNY24)	*MATa, his3Δ1, leu2Δ, met15Δ0, ura3Δ0, cbp4::CBP4-TEV-ProtA-7His-HIS3MX6*	This study
Cbp6^{ProtA} (NNY25)	*MATa, his3Δ1, leu2Δ, met15Δ0, ura3Δ0, cbp6::CBP6-TEV-ProtA-7His-HIS3MX6*	This study
cbp3Δ (NNY26)	*MATα, his3Δ1, leu2Δ, met15Δ0, ura3Δ0, CBP3::HIS3MX6*	This study
cbp4Δ (NNY27)	*MATα, his3Δ1, leu2Δ, met15Δ0, ura3Δ0, CBP4::HIS3MX6*	This study
cbp6Δ (NNY28)	*MATα, his3Δ1, leu2Δ, met15Δ0, ura3Δ0, CBP6::HIS3MX6*	This study

Supplementary Table 2

Oligonucleotides used is this study

Oligo	Sequence	Purpose
NN034	GGATTTAGGTGACACTATAGAATACATGGTTG CATCAGCAAATGCGGG	Forward primer for synthesis of radiolabeled truncated Ina22 (devoid of a presequence)
NN035	GAGCCTACTACATCATCATTAGAAATTTTGGG	Synthesis of radiolabeled $Ina22^{27-216}$ protein
NN036	CTACTACATCATCATGGCGATTTCATTCAAATC ACC	Synthesis of radiolabeled $Ina22^{27-206}$ protein
NN037	CTACTACATCATCATCATCATCATATGGACAT	Synthesis of radiolabeled $Ina22^{27-196}$ protein
NN038	CTACTACATCATCATAACCTTTTCATCTTCTTT TTTC	Synthesis of radiolabeled $Ina22^{27-186}$ protein
NN039	CTACTACATCATCATTAAATCTTTTAGAACATT C	Synthesis of radiolabeled $Ina22^{27-176}$ protein
NN040	CTACTACATCATCATAATCCCTTCTTTGTTCTT AACGC	Synthesis of radiolabeled $Ina22^{27-166}$ protein
NN041	CTACTACATCATCATTGGAGTTTTTTCGATTAC TTC	Synthesis of radiolabeled $Ina22^{27-156}$ protein
NN042	CTACTACATCATCATCTCGTCTATTATACTTTG CC	Synthesis of radiolabeled $Ina22^{27-146}$ protein
NN043	CTACTACATCATCATCAATGACTCTTCCTTAGG AACC	Synthesis of radiolabeled $Ina22^{27-136}$ protein
S1_Ina22	CAACAAGGAAAGACAAGTCATACGTAAAAGGT GTAAGGAAAAATGCGTACGCTGCAGGTCGAC	Forward primer for *INA22* deletion
S2_Ina22	GCGTTATATTTACATGTGGTATATCCGGATGC ATAGAGCCTACTAATCGATGAATTCGAGCTCG	Reverse primer for *INA22* deletion/HA-tagging
S3_Ina22	TTGAATGAAATCGCCAAAGAACATGATAAAAT CCCAAAATTTCTACGTACGCTGCAGGTCGAC	C-terminal HA-tagging of Ina22
NN079	GAAAAAACTCCAGATGCTGGCGTTAAGAACAA AGAAGGGATTGTCACTTAGCGTACGCTGCAG GTCGAC	Generation of $Ina22^{1-168}$ strain
NN081	GTTCTAAAAGATTTAGAAAAGTCGAAAAAAGA AGATGAAAAGGTTTACCTATAGCGTACGCTGC AGGTCGAC	Generation of $Ina22^{1-188}$ strain
NN181	GCTTGCGTTATATTTACATGTGGTATAT-CCGGATGCATAGAGCCTAATCGATGAATTCGA GCTCG	C-terminal FLAG-tagging of Ina22
NN182	GATAAAATCCCAAAATTTCTAGACTACAAAGA CGATGACGACAAGTAGCGTACGCTGCAGGTC GAC	C-terminal FLAG-tagging of Ina22
NN090	GGAAAGCTGATAGTACCGAATTTTTTTTTTTT TGGCACGATATGCGTACGCTGCAGGTCGAC	Generation of *ATP23* overexpression strain
NN093	GCATGGTCCGTCTCCACCACTCAAACCCAGC ATTATCCCCACTGCTATTCATCGATGAATTCTC TGTCG	Generation of *ATP23* overexpression strain
NN091	GCGTCTATATATTTTCTATTATAGAATATTGTC ATTTATTACATTGGTTCAATCGATGAATTCGAG CTCG	C-terminal tagging of Atp23
NN092	GGGACAGTTGCTTCGCCGATACGAGACCGTT TGATGAGATTTACAGACGTACGCTGCAGGTC GAC	C-terminal tagging of Atp23
NN150	GCCAAATAGCCGCCCATCCCTTGTTGGCCGC CGCACAAAGCGTCAACTTCAATCGATGAATTC	C-terminal ProtA-tagging of Atp10

	GAGCTCG	
NN151	GGCTACTCCATCTGAAAAGGAAGCATTGTGGAAGTTTGCCAAACGTCTGCGTACGCTGCAGGTCGAC	C-terminal ProtA-tagging of Atp10
NN185	GGATTTAGGTGACACTATAGAATACATGCAGGGCACTTTTAAAAGGTTTTACCATCCC	Synthesis of radiolabeled Atp10
NN186	TTACATCATCATCAGACGTTTGGCAAACTTCCACAATGCTTCCTTTTC	Synthesis of radiolabeled Atp10
NN222	GATCTCCGTAACAATCTCTTCAGCTATTCCAACACTTGATGCGTACGCTGCAGGTCGAC	Deletion of *CBP3* gene
NN223	CTAAACGAGCTAGTTTGTAACTTCAAAACTTATGAAAACACATCGATGAATTCGAGCTCG	Deletion/C-terminal tagging of *CBP3*
NN224	CCTAAAACCTTACCAAGCGAGAGAAGTAGGCTGTCATATACAAACCGTACGCTGCAGGTCGAC	C-terminal tagging of Cbp3
NN225	GCTCATCCCCCGGGGTATTTTATCAAGATAAAATTTTATATACATGCGTACGCTGCAGGTCGAC	Deletion of *CBP4* gene
NN226	GCAAAAGTTCAAGCTGCCCTTCCTAATTGAGTGACCCGACCCATCTAATCGATGAATTCGAGCTCG	Deletion/C-terminal tagging of *CBP4*
NN227	GGAAATAGTCCAGGATAAGCAGGTTAAAAGCTGGTGGCGCTTCTGGCGTACGCTGCAGGTCGAC	C-terminal tagging of Cbp4
NN228	CAGCTTACCAAGTTAAACTCCGTATTCCACAAGCAAGTGCCAAAATGCGTACGCTGCAGGTCGAC	Deletion of *CBP6* gene
NN229	GAATAAATATGTATTTACAAGCTTAGAAAATAATGTGCTCTTTA ATCGATGAATTCGAGCTCG	Deletion/C-terminal tagging of *CBP6*
NN230	GAAAAAGGAAAGCTTATTTACTGCAATGAGAACTGTATTATTTGGTAAACGTACGCTGCAGGTCGAC	C-terminal tagging of Cbp6
