# Supplementary material for: INA complex liaises the F1Fo-ATP synthase membrane motor modules
Source: Nat Commun. 2017 Nov 1;8:1237. doi: 10.1038/s41467-017-01437-z (PMC5665977; doi:10.1038/s41467-017-01437-z)
Supplement: Supplementary file 2 — Description of Additional Supplementary Files [file 41467_2017_1437_MOESM2_ESM.pdf]

Description of Supplementary Files

File name: Supplementary Data 1
Description: Mass spectrometric analyses of proteins purified together with Atp10, using Atp10^{ProtA} containing mitochondria from wild type and *ina22Δ* cells

File name: Supplementary Data 2
Description: Mass spectrometric analyses of proteins purified together with Atp23 upon immunoisolation of Atp23 from wild type and *ina22Δ* cells

File name: Supplementary Data 3
Description: Mass spectrometric analyses of protein complexes separated by BN-PAGE after immunoisolation of Ina22^{FLAG} from wild type mitochondria
